# Longitudinal impact of asymptomatic malaria/HIV-1 co-infection on *Plasmodium falciparum* gametocyte transcript expression and transmission to *Anopheles* mosquitoes

**DOI:** 10.3389/fcimb.2022.934641

**Published:** 2022-09-16

**Authors:** Ashleigh Roberds, Carolyne Kifude, Janet Oyieko, Stephen Ocholla, James Mutunga, David Oullo, Charles Waga, Zhaozhang Li, Shirley Luckhart, V. Ann Stewart

**Affiliations:** ^1^ Department of Preventive Medicine and Biostatistics, Division of Global Public Health, Uniformed Services University of the Health Sciences, Bethesda, MD, United States; ^2^ Kombewa Clinical Research Center, Kenya Medical Research Institute - United States Army Medical Research Directorate - Africa, Kisumu, Kenya; ^3^ Department of Entomology and Vector Biology, United States Army Medical Research Directorate - Africa/Kenya Medical Research Institute, Kisumu, Kenya; ^4^ Department of Biological Sciences, Mount Kenya University, Thika, Kenya; ^5^ Biomedical Instrumentation Center, Uniformed Services University of the Health Sciences, Bethesda, MD, United States; ^6^ Department of Entomology, Plant Pathology and Nematology and Department of Biological Sciences, University of Idaho, Moscow, ID, United States

**Keywords:** malaria, HIV, co-infection, gametocytes, ddPCR, *plasmodium falciparum*, mosquito infectivity, transmission

## Abstract

Despite significant developments towards malaria reduction, parasite transmission in the common context of HIV-1 co-infection and treatment for one or both infections has not been fully characterized. This is particularly important given that HIV-1 and malaria chemotherapies have the potential to alter gametocyte burden and mosquito infectivity. In this study, we examined 782 blood samples collected from a longitudinal cohort of 300 volunteers with asymptomatic parasitemia seeking HIV testing or treatment in the endemic region of Kisumu, Kenya, to define the impacts of HIV-1-malaria co-infection, antiretroviral therapy (ART) plus trimethoprim-sulfamethoxazole (TS) and the antimalarials artemether/lumefantrine (AL) on *Plasmodium falciparum* gametocyte transcript prevalence and parasite transmission to the African malaria mosquito *Anopheles gambiae*. Volunteers were assigned to three distinct HIV-1 groups: HIV-1 positive on treatment, HIV-1 positive newly diagnosed, and HIV-1 negative. Volunteers were monitored monthly over the course of six months. Using our highly sensitive digital droplet PCR (ddPCR) assay of three gametocyte specific transcript markers, we detected gametocyte transcripts in 51.1% of *18S* positive volunteers across all study groups and time points. After correcting for multiple comparisons, the factors of HIV-1 status, time, CD4+ T-cell levels and hematocrit were not predictive of gametocyte prevalence or transmission. However, among those volunteers who were newly diagnosed with HIV-1 and malaria positive by rapid diagnostic test (RDT) at enrollment, the initiation of ART/TS and AL treatment was associated with a significant reduction in gametocyte transcript prevalence in the subsequent month when compared to HIV-1 negative volunteers treated with AL. To assess gametocyte transmissibility, volunteer blood samples were used in standard membrane feeding assays (SFMA) with laboratory-reared *A. gambiae*, with evidence of transmission confirmed by at least one of 25 dissected mosquitoes per sample positive for at least one midgut oocyst. HIV-1 status, CD4+ T-cell levels and hematocrit were not significantly associated with successful transmission to *A. gambiae.* Analysis of SMFA blood samples revealed that 50% of transmission-positive blood samples failed to test positive by *Plasmodium*-specific *18S* ribosomal RNA quantitative PCR (qPCR) and 35% failed to test positive for any gametocyte specific transcript marker by droplet digital (ddPCR), documenting that transmission occurred in the absence of molecular parasite/gametocyte detection. Overall, these findings highlight the complexity of HIV-1 malaria co-infection and the need to further define the unpredictable role of asymptomatic parasitemia in transmission to mosquitoes.

## 1 Introduction

In areas of high endemicity for malaria, such as sub-Saharan Africa, high prevalence of asymptomatic malaria parasitemia poses a risk to malaria elimination efforts. Many of the same geographical areas represent a significant portion of the world’s HIV-1 infections, leading to higher probability of HIV-1-malaria co-infection. It is well described that HIV-1-malaria confection increases clinical malaria and parasitemias in adults (Hewitt et al., 2006; Flateau et al., 2011). However, less is known about the impact that HIV-1 co-infection may have gametocyte burden and malaria parasite transmission, specifically in asymptomatic populations [reviewed in (Roberds et al., 2021)]. In a rhesus macaque model of simian immunodeficiency virus (SIV) malaria co-infection, co-infection was significantly associated with increased gametocytemia and parasite transmission to *Anopheles freeborni* mosquitoes (Trott et al., 2011). In 2020, an epidemiological study investigating the impact of HIV-1 co-infection on the prevalence of asymptomatic gametocytemia suggested that there is an increased risk for gametocyte prevalence amongst HIV-1 positive individuals (Stiffler et al., 2020). These observations prompted questions regarding the biological complexities surrounding both diseases and parasitic responses to the changes in intrinsic host factors and initiation of chemotherapies over time.

There are few studies that have examined the clinical parameters of HIV-1-malaria co-infection and their association with parasite transmission. Anemia is of particular interest in that it is observed independently in both malaria and HIV-1 infections (Belperio and Rhew, 2004) and has been associated with altered gametocytogenesis and gametocyte development [reviewed in (Bousema and Drakeley, 2011)]. Recent studies have shown that asymptomatic malarial parasitemias were significantly associated with abnormal hematological outcomes in people living with HIV-1 and co-infected with malaria (Kamau et al., 2020), suggesting that parasite transmission could be altered in the context of suppressed immunity and antiretroviral therapy (ART). The effects of immune deficiency on malarial parasitemia and clinical episodes have been well studied [reviewed in (Hewitt et al., 2006; Flateau et al., 2011)]. Due to the lack of significant clinical association with gametocytes in endemic populations, there have been fewer studies that have investigated the effects of immunodeficiency on gametocytes and parasite transmission.

Antifolate therapy, such as trimethoprim-sulfamethoxazole (TS) is often prescribed to HIV-1 infected individuals for prophylaxis for opportunistic infections. Effects of TS on gametocytes and gametocytemia have been notable. For example, gametocytemia was observed to increase almost immediately after drug initiation and peaked around two weeks into therapy (Bousema et al., 2006). Because TS does not kill gametocyte-committed rings, these results could be due to the continued development of early gametocytes. For example, Hobbs et al. reported that TS reduced the risk of peripheral parasitemia and oocyst infection in exposed mosquitoes but did not reduce gametocyte viability or inhibit gametocyte exflagellation (Hobbs et al., 2012; Hobbs et al., 2013). The contrasting results between gametocyte burden and transmission success highlight the need to include mosquito feeding assays when quantifying parasite transmission potential in epidemiological studies (Ouédraogo et al., 2009; Bousema et al., 2012) and clinical studies (Sawa et al., 2013).

Results from our prior point-prevalence study (Stiffler et al., 2020) revealed that HIV-1 was associated with increased prevalence and abundance of *Plasmodium falciparum* gametocyte-specific transcripts in asymptomatic adults in western Kenya. Given that chronic asymptomatic *P. falciparum* parasitemia has been associated with long term intermittency of gametocytemia (Smalley et al., 1981) and that asymptomatic infections have been associated with low quantities of gametocytes [reviewed in (Roberds et al., 2021)], we sought to investigate the longitudinal impact of clinical parameters and initiation of chemotherapies on *P. falciparum* gametocyte burden and subsequent parasite transmission to the *Anopheles gambiae* using highly sensitive digital droplet PCR (ddPCR) and standard membrane feeding assays (SMFA).

## 2 Material and methods

### 2.1 Sample collection and study design

A total of 300 study participants were voluntarily enrolled from study sites in Kisumu County, Kenya, after self-presenting for HIV testing at satellite HIV Testing and Counseling (HTC) Centers or the HTC at Kombewa Sub-County Hospital (KCH). All participants were apparently healthy adults with asymptomatic *Plasmodium falciparum* parasitemia. Participants were screened and enrolled until a target of approximately 100 participants was met in each of the target categories: HIV-1 negative, HIV-1 positive newly diagnosed, and HIV-1 positive on ART and TS (Oyieko et al. under review). Additional eligibility included willingness and availability for blood sampling for six months of monthly follow up visits. Initial invitation for study participation was presented to patients during HTC counseling or clinic visits with a follow-on opportunity to attend a detailed information session and provide informed consent. Enrolled patients who were stable on ART and TS were treated in accordance with guidelines from the Kenyan Ministry of Health (MoH). Whole blood samples were collected in accordance with Institutional Review Board protocols and with signed informed consent to include six months of follow-up visits. Rapid diagnostic tests (RDTs) were used for HIV-1 and malaria screening and confirmation followed by blood collection for further clinical and molecular testing (Oyieko et al., 2022, manuscript submitted for publication). Malaria RDTs and blood collection for molecular testing were performed at each clinic visit for all study participants regardless of HIV-1 positivity and treatment status. Any patients with positive malaria RDT results were administered a three-day regimen of AL (Artemether-Lumefantrine) in accordance with Kenya MoH guidelines (Ministry of Health, 2016). Newly diagnosed HIV-1 participants were immediately started on ART, multivitamin supplementation, and TS. Additional blood was collected for a Standard Membrane Feeding Assay (SMFA) in a subset of volunteers described below. Polymerase Chain Reaction (qPCR) assays were used for research-based questions to include the foundational testing for the presence of *Plasmodium*-specific transcripts using *18S* ribosomal RNA and quantitative PCR (qPCR) (Kamau et al., 2020; Kifude et al., 2021) (Kifude et al. under review). Samples positive by *18S* qPCR were subjected to gametocyte transcript analysis.

Blood collected for molecular analysis was blotted onto Whatman^®^ 903 Protein saver filter paper cards (GE Healthcare Life Sciences, Chicago, IL USA) and stored at -80°C until processed for RNA and DNA extraction (Jones et al., 2012; Pritsch et al., 2012). Sample collected occurred between August 2018 and May 2020. RNA and DNA extraction for gametocyte analysis occurred between August 2019 and April 2021. In brief, a single air-dried 50 μL blood spot (DBS) was minced and nucleic acids were extracted using the RNeasy Mini Kit (Qiagen, Hilden, Germany) according to the manufacturer’s standard protocol for RNA extraction. Genomic DNA was digested as part of this procedure prior to the reverse transcription of RNA. cDNA synthesis was conducted using the QuantiTect Reverse Transcription Kit (Qiagen, Hilden, Germany) and following the manufacturers standard protocol.

### 2.2 Selection of gametocyte specific transcripts

Target genes were selected using documented transcript levels of genes from purified male and female *P. falciparum* gametocytes (Lasonder et al., 2016). *Pfs16 (Plasmodium falciparum* parasitophorous vacuole membrane protein S16, Pf3D7_0406200) was selected as an early molecular marker of gametocytes (Bruce et al., 1994; Dechering et al., 1997; Berry et al., 2009) showing high transcript levels with relatively equal female to male ratio of 1.28:1.00 (Lasonder et al., 2016). *PfMGET (Plasmodium falciparum* male gametocyte-enriched transcript, Pf3D7_1469900) was shown to have more abundant gene transcripts than other previously used male markers (Stone et al., 2017), and *Pfs25 (Plasmodium falciparum* ookinete surface protein P25, Pf3D7_1031000) was selected as an abundantly expressed female-specific marker in stage V gametocytes (Schneider et al., 2004; Lasonder et al., 2016). *Pfs16* and *Pfs25* primers and probes (**Supplementary Table 1**) were derived and adapted for use with digital droplet PCR (ddPCR) from Stiffler et al. (Stiffler et al., 2020), and *PfMGET* primers and probe (**Supplementary Table 1**) were derived and adapted for use with ddPCR from Wang et al. (Wang et al., 2020).

### 2.3 Digital droplet PCR

A duplex ddPCR assay was adapted using separate indicators for *PfMGET* and *Pfs25* markers in a single reaction, and *Pfs16* was quantified in a separate reaction (Pomari et al., 2020). For both assays, each reaction included 11 μL Supermix for Probes (no dUTPs) (Bio-Rad Laboratories Inc (2018)., Hercules, CA, US), 2 μL cDNA template, nuclease free water as needed, 455 nM of each primer and 250 nM of each probe in a final reaction volume of 22 μL. Droplets were generated using the QX200 Droplet Generator (Bio-Rad Laboratories Inc (2018), Hercules, CA, US) within a range of 10,000 to 20,000 droplets. Reactions were performed on the C1000 Touch Thermal Cycler (Bio-Rad Laboratories Inc (2018), Hercules, CA, US) under the following programs (ramp rate 2°C/second): Duplex *PfMGET*/*Pfs25* - 95°C for 10 minutes, 40 cycles of 94°C for 30 seconds and 50°C for 1 minute, 98°C for 10 minutes; *Pfs16* - 95°C for 10 minutes, 40 cycles of 94°C for 30 seconds and 55°C for 1 minute, 98°C for 10 minutes. Droplets were analyzed and counted on a QX200 Droplet Reader (Bio-Rad Laboratories Inc (2018), Hercules, CA, US).

#### 2.3.1 ddPCR assay optimization and validation

Each marker of interest was cloned into the PCR 2.1 TOPO TA Vector (Life Technologies, Carlsbad, CA, USA) in accordance with manufacturers’ protocols (Invitrogen TOPO TA Cloning Kit with pCR 2.1-TOPO, One Shot TOP10 Chemically Competent *E. coli*). Plasmid DNA was purified using the QIAprep Spin MiniPrep kit (Qiagen, Hilden, Germany), quantified using a spectrophotometer (NanoDrop 2000C, ThermoFisher Scientific, Waltham, MA, USA), and verified by Sanger sequencing by GENEWIZ from Azenta Life Sciences (South Plainfield, NJ, USA). Ten-fold serial dilutions of each plasmid ranging from 10,000 copies per μL to 0.1 copies per μL were generated by diluting in water. Additional two-fold dilutions between 10 copies per μL and 0.3125 copies per μL were used to determine lower limits of detection and quantification. The limits of detection and quantification for *Pfs25*, *PfMGET*, and *Pfs16* were 3.125, 6.25, and 1.00 copies/μL respectively. The limit of detection was defined by the presence of at least one positive droplet above the statistically calculated threshold. Temperature gradient protocols were used to identify optimal annealing temperatures. Additional optimization was conducted to determine the greatest separation of relative fluorescence units (RFU) between control positive and negative droplets.

#### 2.3.2 ddPCR analysis

For the duplex assay, a data-driven approach was used to identify the threshold between positive and negative droplets using no-template-control (NTC) wells within each ddPCR plate. The ddpcRquant approach was adapted and used to determine the number of positive droplets in the duplex assay (Trypsteen et al., 2015). For the *Pfs16* single assay, droplets were considered positive if they were above 4000 RFU and the NTC negative droplets averaged between 1500 and 3000 RFU.

ddPCR concentration (as copies of target per microliter of final ddPCR reaction) was manually calculated using a Poisson algorithm, where [Concentration = - ln (Negative droplets/Total number of droplets)/Volume of Droplet] and the volume of droplet was always 0.85 nL. Concentration was further translated into total copies of target per microliter of starting sample using the following formula: [(Concentration x Ratio of total PCR volume (22 μL) to sample volume (2 μL)) x original sample volume (2 μL)] (Bio-Rad Laboratories Inc (2018)).

### 2.4 Sampling for mosquito infectivity study

A subset of 100 volunteers was recruited and transported to the Kenya Medical Research Institute (KEMRI)/U.S. Army Medical Research Directorate-Africa (USAMRD-A) Entomology Department laboratories in Kisian, Kenya. One mL of blood was collected from each volunteer at each visit for SMFA using laboratory-reared adult female *A. gambiae*. Given that gametocytes circulate in the periphery for an average of 3.4-6.4 days (Bousema and Drakeley, 2011), we included samples for clinical analysis that were within two days of SMFA. Accordingly, bloods samples for molecular analyses from each volunteer were collected on the date of the SFMA or within two days before or after the SMDA. Based on these parameters, blood samples from 44 HIV-1 positive volunteers and 39 HIV-1 negative volunteers were screened by qPCR, ddPCR, and transmission to *A. gambiae* by SMFA.

#### 2.4.1 Insectary procedures for mosquito rearing

SMFAs were conducting using laboratory-reared *A. gambiae s.s.*, originally obtained from Luanda village, Kisumu County Western Kenya. The mosquitoes were reared in the USAMRD-A/K Insectary at KEMRI Centre for Global Health in Kisian, Kisumu. Adult mosquitoes were kept in 30x30x30 cm gauze-covered cages under ambient conditions. They were maintained on a 10% sterile sucrose solution and provided with water on cotton pads to increase relative humidity in the cages. Cow blood for colony mosquito feeding was obtained freely from local abattoirs; raw blood was collected from freshly slaughtered cow’s jugular vein and anticoagulated with EDTA 60 mg/L for short-term (7-10 days) refrigerated storage. Mosquitoes were fed on cow blood through a Hemotek membrane feeder for 30 minutes and maintained under adult rearing conditions as previously described. After two days, an oviposition cup was placed in the previously blood-fed cage; any eggs laid on the moist filter paper were transferred into rearing pans with approximately 3 liters of old rainwater. The larvae were fed 2-3 times a day on Tetramin^®^ fish food and Brewer’s yeast; they were maintained at a temperature of 27 ± 1°C until pupation. Water was replaced every other day to maintain good larval growth. Adult mosquitoes were kept inside a separate room, where temperatures were maintained at 27°C and relative humidity at 70–90% and were fed on 10% sterile sucrose solution. The insectary was set to a photoperiod of 12 hours darkness and 12 hours light.

#### 2.4.2 Standard membrane feeding assay and mosquito dissection

Laboratory-reared, 3–5-day old *A. gambiae* were partially starved for 3-5 hours prior to SMFA. They were subsequently allowed to feed for 20 minutes *via* an artificial membrane attached to a water-jacketed glass-bell parafilm membrane feeder maintained at 37°C. In accordance with the SMFA protocol (Ouédraogo et al., 2013), a 1 mL sample of the volunteer’s blood was drawn into pre-heated glass feeders. After the SFMA, fully engorged mosquitoes were transferred to environmental chambers with automated control of temperature and humidity (27°C, 80% respectively) and a fixed light-dark cycle (12h/12h). At 10 days post-feeding, samples of 25 mosquitoes per SFMA were moved from the environmental chambers to a freezer (-20°C) for immobilization prior to washing with 70% ethanol and rinsing with Phosphate Buffer Solution (PBS). Oocyst counting has been determined to be an accurate readout for transmissions success, even from low-intensity infections (Stone et al., 2013). Accordingly, mosquitoes were dissected as described by (Ouédraogo et al., 2013) for this purpose. After dissection and staining in 1% mercurochrome for 10 minutes, individual mosquito midguts were placed on a microscope slide with a cover slip and transferred to a compound microscope for enumeration of oocysts on the midgut. DBS corresponding to oocyst-positive mosquitoes were also included in gametocyte transcript analyses regardless of *18S* results.

### 2.5 Statistical methods

Data were analyzed using R (R Core Team, under review, Vienna, Austria), SPSS (IBM Corp, Armonk, NY, USA) and GraphPad Prism 9 (GraphPad, San Diego, CA, USA). Samples were considered gametocyte positive if they were positive for at least one gametocyte-specific transcript. Samples tested for transmission were considered positive if at least one oocyst was detected in one midgut in a single group of mosquitoes used for a single SFMA. Gender, age, and differences between study groups at baseline were compared by Pearson’s chi-square test. Proportional analyses were also conducted using Pearson’s chi-square test throughout the study. When comparing markers from *18S* positive individuals with markers from those who tested negative for *18S* by qPCR, Mann Whitney non-parametric tests was used. Based on repeated measures in our design, generalized estimating equations (GEE) were used to quantify absolute differences across time and to generate adjusted odds ratios. In instances of log transformation of *18S* qPCR data and gametocyte transcript ddPCR data, undetected samples were defined as “0.01” for transformation. Holm’s method was used to adjust necessary p-values for multiple comparisons. The level of significance was set at alpha = 0.05.

## 3 Results

### 3.1 Study profile

**Figure 1:**
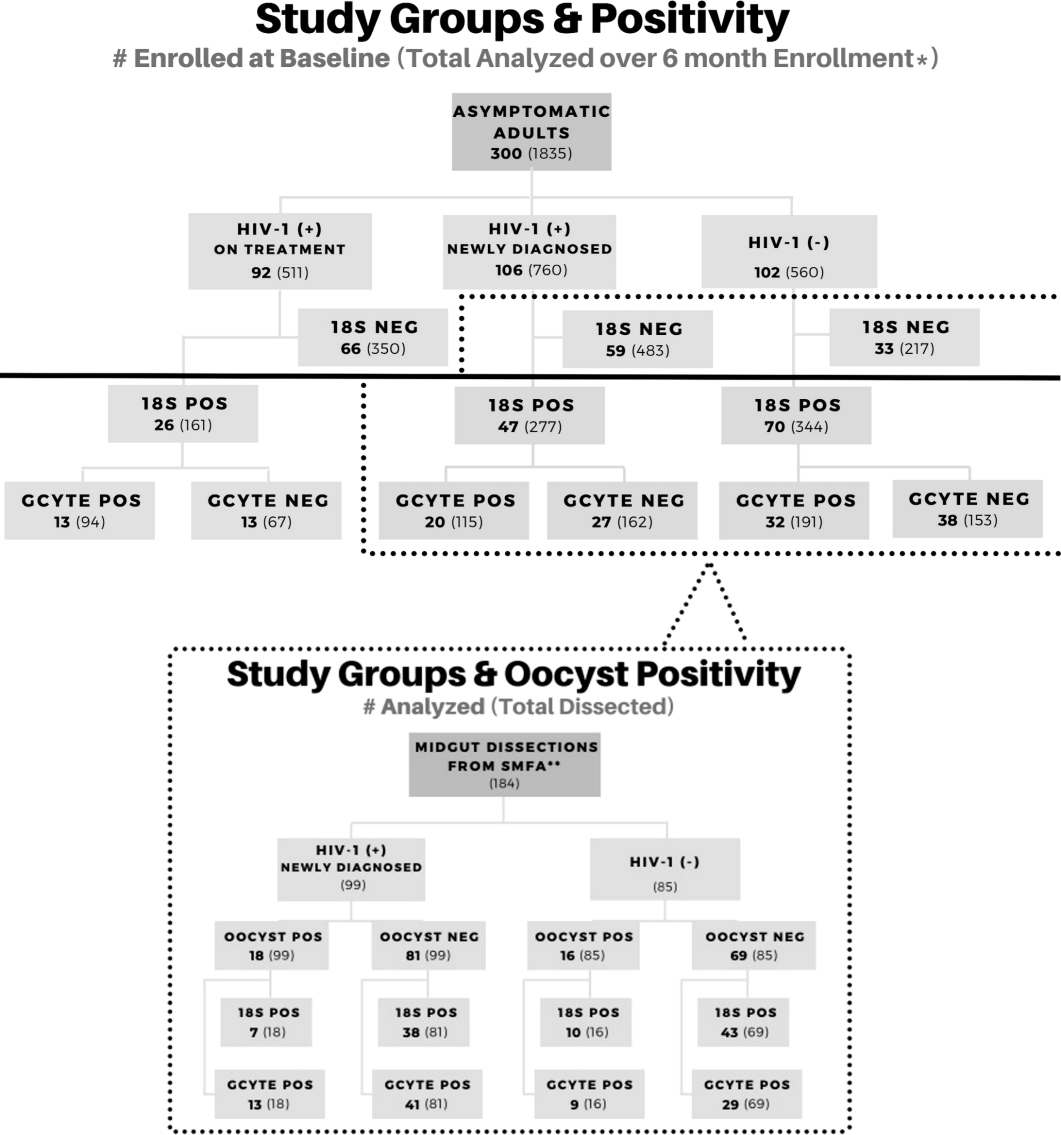
Study Profile Overview. Participant enrollment by study group and corresponding molecular data for asexual and sexual parasite biomarkers. Groups below the solid black line were included in this study for gametocyte analysis. A subset of volunteers was enrolled in a transmission study using the Standard Membrane Feed Assay (dotted line). Positive samples are abbreviated as (+) or POS. Negative samples are abbreviated as (-) or NEG.

A total of 300 screened participants were enrolled for this study: 102 HIV-1- negative individuals and 198 HIV-1 positive individuals. HIV-1 positive individuals were further categorized as 106 HIV-1 positive newly diagnosed at enrollment and 92 HIV-1 positive and stable on ART and TS (**Figure 1**). A total of 782 unique samples from all participants during all time points (two lost in transport) were determined to be *18S-*positive by qPCR and included in this study; *18S*-negative samples were not analyzed further unless blood fed mosquitoes became oocyst positive. Analysis of *18S* prevalence by category over time are presented in **Supplementary Figure A** (Kifude et al., 2022, manuscript in preparation) and briefly described below. Malaria prevalence by RDT at baseline (month 0, enrollment) was 17.3% (52/300; 22 HIV-1 negative, 26 HIV-1 positive newly diagnosed, 2 HIV-1 positive on treatment) and malaria prevalence across the entire study period by *18S* qPCR was 42.7% (784/1835) with equal prevalence across monthly visits. A larger proportion of HIV-1 negative volunteers was malaria *18S* positive (61.43% *p=0.0156)* compared to the HIV-1 positive volunteers (newly diagnosed 36.45%; on treatment 31.51%). Of note, there was a significant drop in parasitemia, expressed as *18S* copy numbers per microliter, upon initiation of ART and TS from day of enrollment to one-month post-treatment/post-prophylaxis initiation in newly diagnosed HIV-1 positive volunteers (Kifude et al., 2022, manuscript in preparation). Parasitemias were significantly higher among HIV-1 negative individuals throughout the course of the study. As expected, baseline CD4+ T cell levels across all three study groups were consistent with known progression in HIV-1 infection under treatment; CD4+ T cell levels were highest in HIV-1 negative and lowest in the HIV-1 positive newly diagnosed (Oyieko et al., 2022, manuscript submitted for publication).

### 3.2 Baseline and overall characteristics

At baseline (month 0, enrollment), 65/143 (45.5%) *18S-*positive volunteers were positive for at least one gametocyte-specific marker (*Pfs25*, *PfMGET*, or *Pfs16*) by ddPCR. The impact of any seasonality could not be assessed as enrollment spanned 15 months and the study period crossed multiple seasons (**Supplemental Figures A-C**). Baseline prevalences of gametocyte positivity by study group were 46% in HIV-1 negative, 43% in HIV-1 positive newly diagnosed, and 50% in HIV-1 positive on ART and TS. There were no significant differences in gametocyte prevalence among study groups at baseline (Pearson’s Chi-square: *p=*0.827). Across all groups, study participants were divided into age brackets by interquartile ranges (18-23, 24-31, 32-36, 37-59). There were no significant differences in gametocyte prevalence among age groups or by gender at baseline (Pearson’s Chi-square: *p=*0.699, *p=*0.904 respectively). Of the 263 volunteers who were *18S*-positive at any time point, 192 were positive for at least one gametocyte-specific marker at any time point. Of these 192, 54.2% were female and 45.8% were male with a mean age of 32 (range, 18-56).

### 3.3 GEE analysis to compare longitudinal gametocyte-specific transcript prevalence between and within study groups

**Figure 2:**
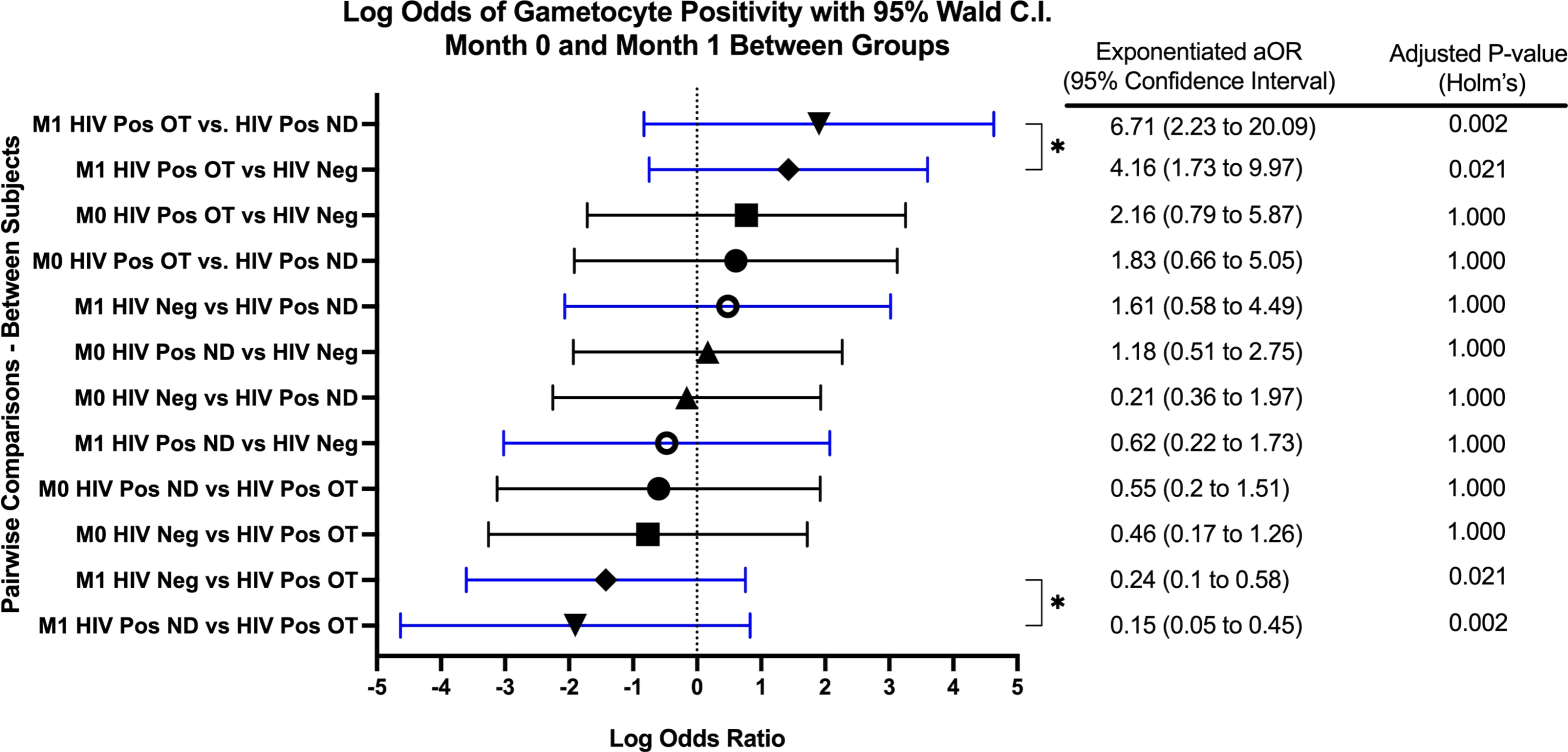
Forest Plot of the Log Odds of Gametocyte Positivity in Month 0 and Month 1. The log odds and accompanying exponentiated adjusted odds ratio of being positive for at least one gametocyte specific transcript between each study group in month 0 (M0) and in month 1 (M1). “HIV Neg” represents HIV-1 negative volunteers. “HIV Pos OT” represents HIV-1 positive volunteers on treatment. “HIV Pos ND” represents HIV-1 positive newly diagnosed volunteers. P-values were adjusted using the Holm’s method for multiple comparisons. Log odds are arranged in descending order. Blue confidence interval lines are comparisons within month 1 and black confidence interval lines are comparisons within month 0.

GEE analysis revealed that the odds of gametocyte specific transcript prevalence were significantly higher in month 1 for HIV-1 positive volunteers on treatment compared to HIV-1 positive newly diagnosed volunteers (aOR: 6.72, adjusted *p*=0.002) and HIV-1 negative volunteers (aOR: 4.16, adjusted *p=*0.021) (**Figure 2**). There were no significant differences among the study groups at any other time point.

GEE analysis also revealed no difference in the odds of gametocyte prevalence when compared to baseline or between any other visits for HIV-1 positive volunteers on treatment and HIV-1 positive newly diagnosed volunteers. For HIV-1 negative volunteers, the odds of being gametocyte positive were significantly higher for months 4 and 5 when compared to month 1 (month 4 aOR: 1.51, *p=0*.002; month 5 aOR: 1.39, *p=0*.040).

Overall, there was no significant interaction effect (adjusted *p=*0.552) of study group and time. The main effect of study group (irrespective of time) was not a significant predictor of gametocyte positivity (adjusted *p*=0.088). Additionally, the main effect of time (irrespective of study group) was also not a significant predictor of gametocyte positivity (adjusted *p*=0.112).

### 3.4 *18S* copy number is the only predictor of gametocyte-specific transcript prevalence

In an effort to predict overall gametocyte positivity, selected clinical and diagnostic predictors were used in the GEE analysis. Predictors included common indicators of altered immune systems based on white blood cell (WBC) (x 10^9^/L) and CD4+ T-cell count, anemia based on red blood cell count (RBC) (x 10^12^/L), age, gender, *18S* copy number/μL, *Pfs25* ddPCR concentration, and *PfMGET* ddPCR concentration. Log transformed *18S* copy number/μL was the only statistically significant predictor for gametocyte prevalence (aOR: 1.202, adjusted *p=0*.018) (**Table 1**). For every 10-fold increase in *18S* copy numbers, the odds of gametocyte positivity increased by 20%.

**Table 1:** GEE Omnibus Results.

Predictor of Gametocyte positivity	Exponentiated aOR	95% Wald Confidence Interval	Adjusted p-value (Holm’s)
Gender (Male : Female)	0.720	0.586 to 1.446	1.000
Time	–	–	0.112
Study Group	–	–	0.088
Age	0.991	0.966 to 1.017	1.000
CD4+ T cells	1.001	1.000 to 1.001	0.575
WBC	0.971	0.860 to 1.096	1.000
RBC	0.955	0.668 to 1.352	1.000
*Log *18S* Copy numbers/μL	1.202	1.069 to 1.352	0.018

Predicting overall positivity using gender (male to female), time (visit month), study group, age, CD4+ T cell levels, white blood cell (WBC) counts (x 10^9^/L), red blood cell (RBC) counts (x 10^12^/L), and log *18S* copy numbers/μL. Time and study group were the main effects in the model, so a p-value only was provided for the Omnibus test. *When log transforming *18S* copy numbers/μL, undetermined results were defined as “0.01” for the transformation.

### 3.5 Sex-specific gametocyte transcripts in each study group over time

**Figure 3:**
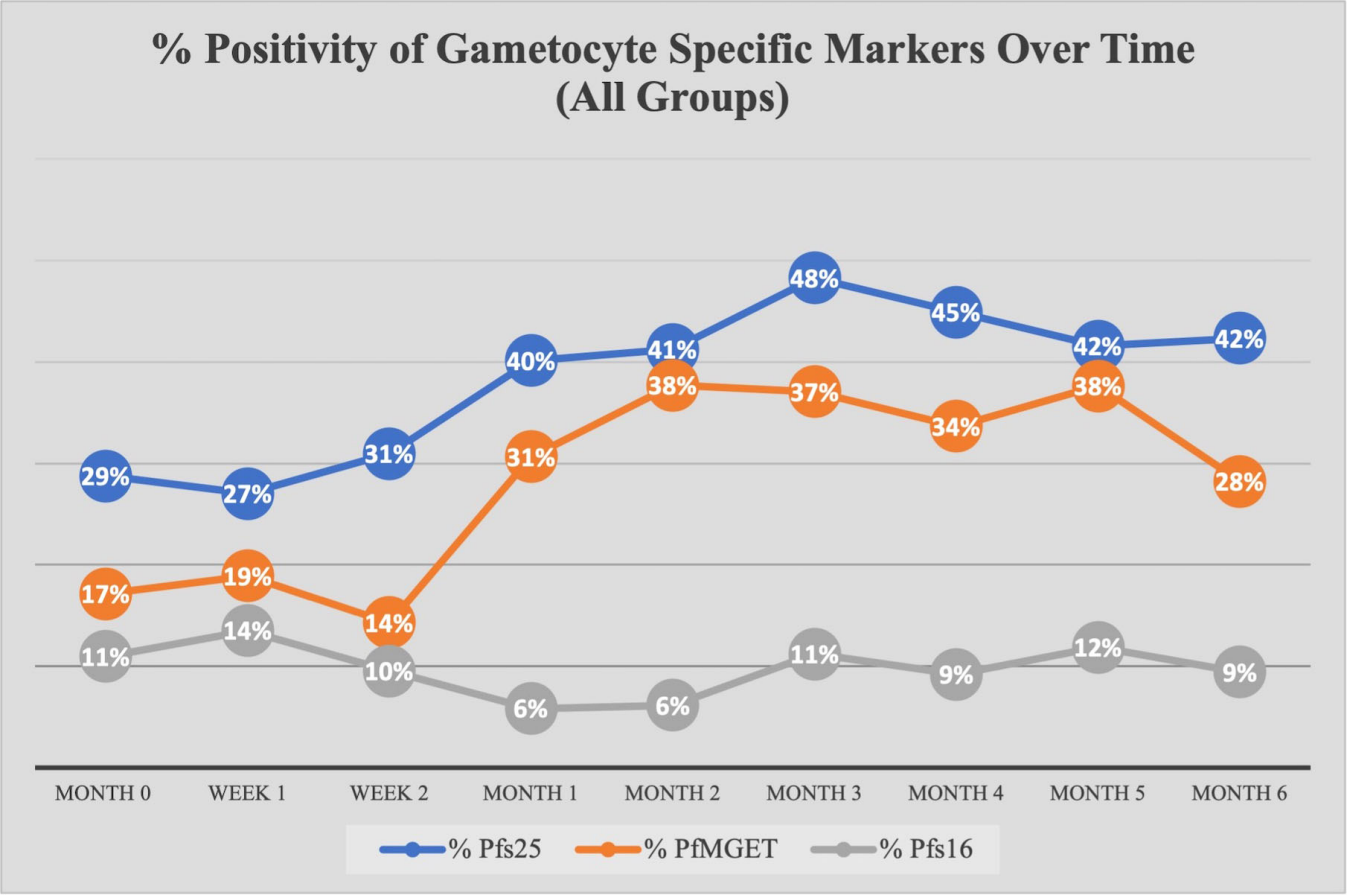
Percent Positivity of Each Gametocyte-Specific Marker Over Time for All Study Groups. The prevalence of each gametocyte-specific transcript biomarker (as a percentage of total analyzed) at each time point/visit.

Across all time points, 782 *18S*-positive samples were analyzed for gametocytes. 400 of 782 samples (51.1%) were positive for at least one gametocyte-specific marker with the majority positive for *Pfs25* (female) and *PfMGET* (male) (**Supplemental Figure D**). Across all groups, there was an increase in the prevalence of *Pfs25* and *PfMGET* transcripts at month 1, while *Pfs16* transcripts remained expressed at low levels (**Figure 3**).

#### 3.5.1 *Pfs25* positive transcripts and ddPCR concentrations

**Figure 4:**
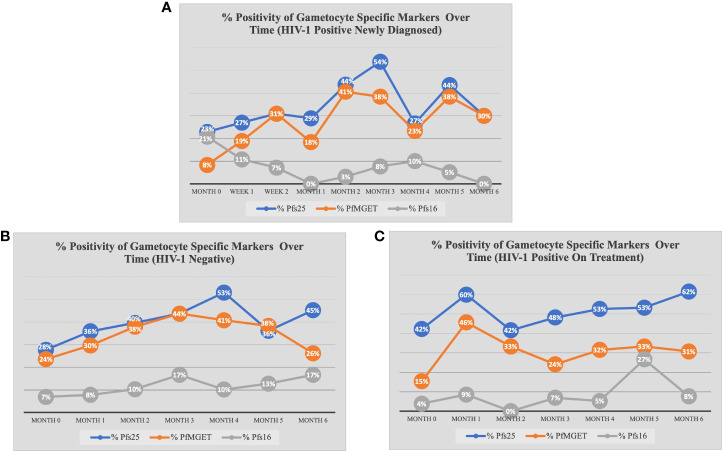
Percent Positivity of Each Gametocyte-Specific Marker Over Time for Each Study Group. The prevalence of each gametocyte-specific transcript biomarker (as a percentage of total analyzed) at each time point for **(A)** HIV-1 positive newly diagnosed volunteers **(B)** HIV-1 negative volunteers and **(C)** HIV-1 positive volunteers on treatment.

At baseline, there were no differences in the odds of being *Pfs25* positive between study groups at any other month besides month 1 (GEE analysis). Similar to overall gametocyte prevalence, the odds of *Pfs25* positivity increased in month 1 for HIV-1 positive on treatment compared to HIV-1 negative and compared to HIV-1 positive newly diagnosed (aOR 5.00, adjusted p-value <0.001; aOR 5.93, adjusted p-value 0.005, respectively). As seen in **Figure 4C**, there was an increase from 42% positive for *Pfs25* in month 0 to 60% positive for *Pfs25* in month 1 within the HIV-1 positive on treatment group. *Pfs25* positivity unexpectedly dropped at month 4 for HIV-1 positive newly diagnosed, but the decreased odds were not significant after adjusting for multiple comparisons.

**Figure 5:**
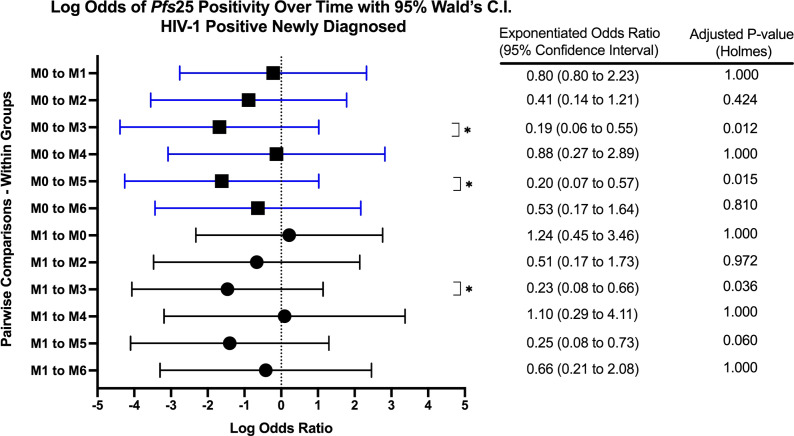
Forest Plot of the Log Odds of *Pfs25* Positivity in HIV-1 Positive Newly Diagnosed Volunteers. The log odds and accompanying exponentiated adjusted odds ratio of being positive for at least one gametocyte-specific transcript within the HIV-1 positive newly diagnosed study group between month 0 (M0) and between month 1 (M1). P-values were adjusted using the Holm’s method for multiple comparisons. Blue confidence interval lines are comparisons with month 0 and black confidence interval lines are comparisons with month 1.

**Figure 6:**
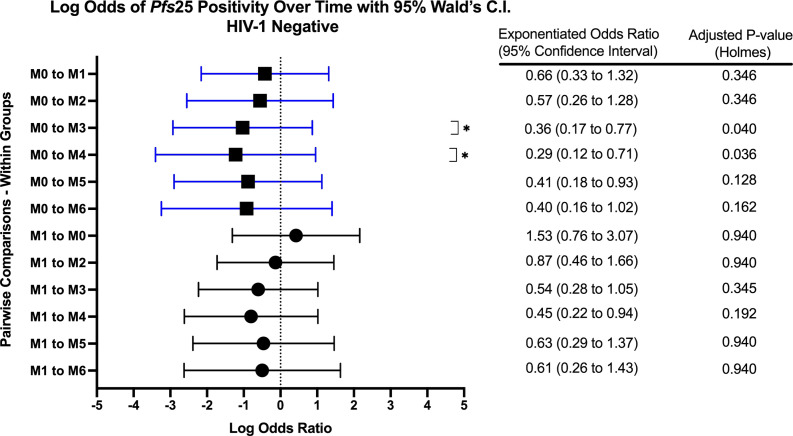
Forest Plot of the Log Odds of *Pfs25* Positivity in HIV-1 Negative Volunteers. The log odds and accompanying exponentiated adjusted odds ratio of being positive for at least one gametocyte-specific transcript within the HIV-1 negative study group between month 0 (M0) and between month 1 (M1). P-values were adjusted using the Holm’s method for multiple comparisons. Blue confidence interval lines are comparisons with month 0 and black confidence interval lines are comparisons with month 1.

When comparing within each group over time, there were no differences in *Pfs25* positivity between timepoints for HIV-1 positive on treatment. HIV-1 positive newly diagnosed exhibited a tendency for increased positivity at months 3 and 5 when compared to baseline and month 1 (**Figure 5**). These observations were similar to those for HIV-1 negative volunteers who exhibited a tendency for increased positivity at months 3 and 4 when compared to baseline (**Figure 6**).

After adjusting for multiple comparisons there were no differences in *Pfs25* ddPCR concentration (**Supplemental Figure E**) between groups at each time point or over time within any group. Contrary to the prevalence analysis, log transformed *18S* copy number did not predict *Pfs25* ddPCR concentration (log transformed). There were no differences in *Pfs25* ddPCR concentrations by gender or age nor any predictive associations between WBC, RBC, or CD4+ T-cell levels.

#### 3.5.2 *PfMGET* positive transcripts and ddPCR concentrations

At baseline, the odds of *PfMGET* positivity did not differ between study groups at any month except for month 1 (GEE Analysis). As with *Pfs25* transcript positivity, the odds of *PfMGET* transcript positivity in HIV-1 positive volunteers on treatment were higher at month 1 when compared to HIV-1 negative volunteers and HIV-1 positive newly diagnosed volunteers (aOR 3.08, adjusted p-value 0.046 and aOR: 7.33, adjusted p-value 0.009, respectively).

**Figure 7:**
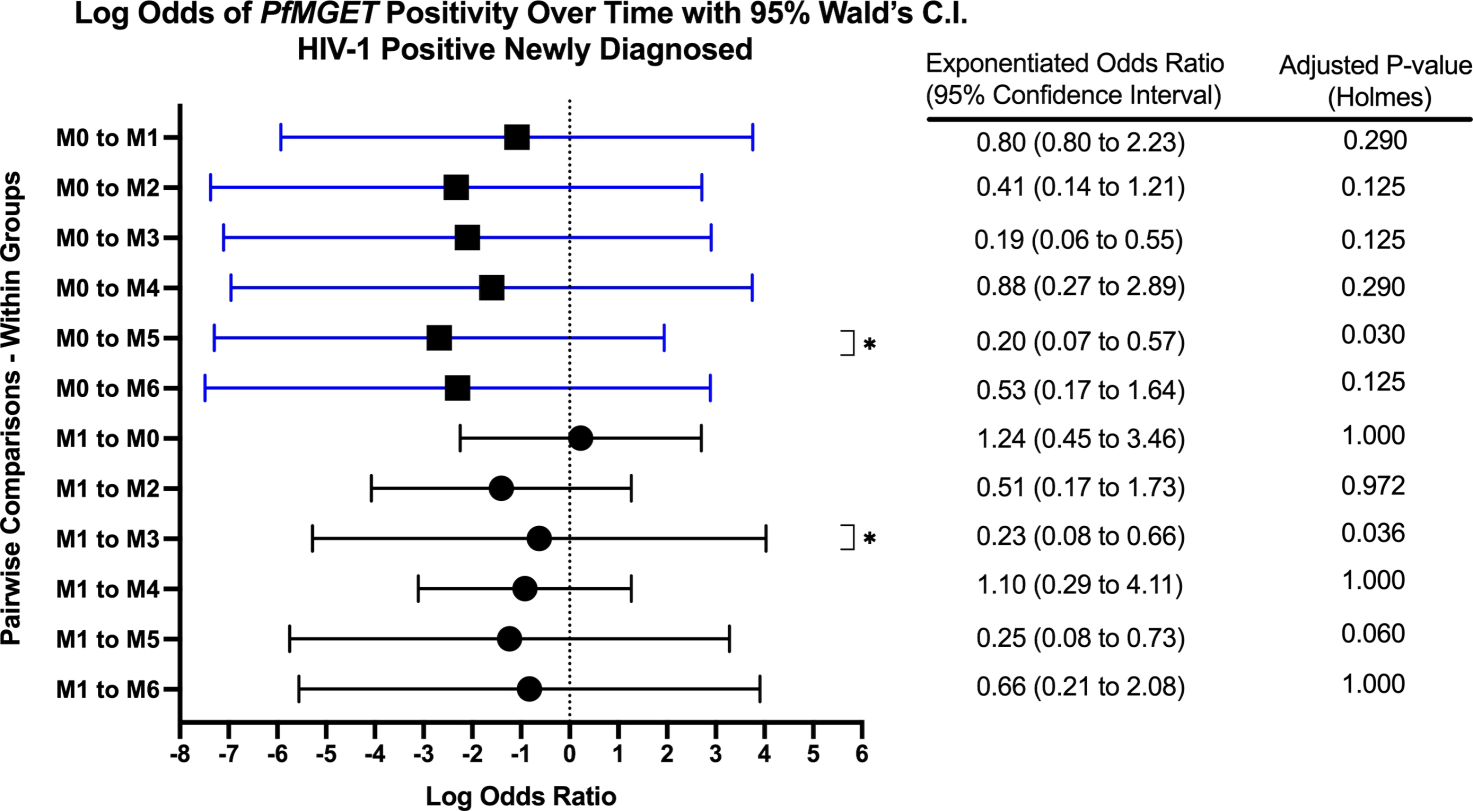
Forest Plot of the Log Odds of *PfMGET* Positivity in HIV-1 Positive Newly Diagnosed Volunteers. The log odds and accompanying exponentiated adjusted odds ratio of being positive for at least one gametocyte specific transcript within the HIV-1 positive newly diagnosed study group between month 0 (M0) and between month 1 (M1). P-values were adjusted using the Holm’s method for multiple comparisons. Blue confidence interval lines are comparisons with month 0 and black confidence interval lines are comparisons with month 1.

**Figure 8:**
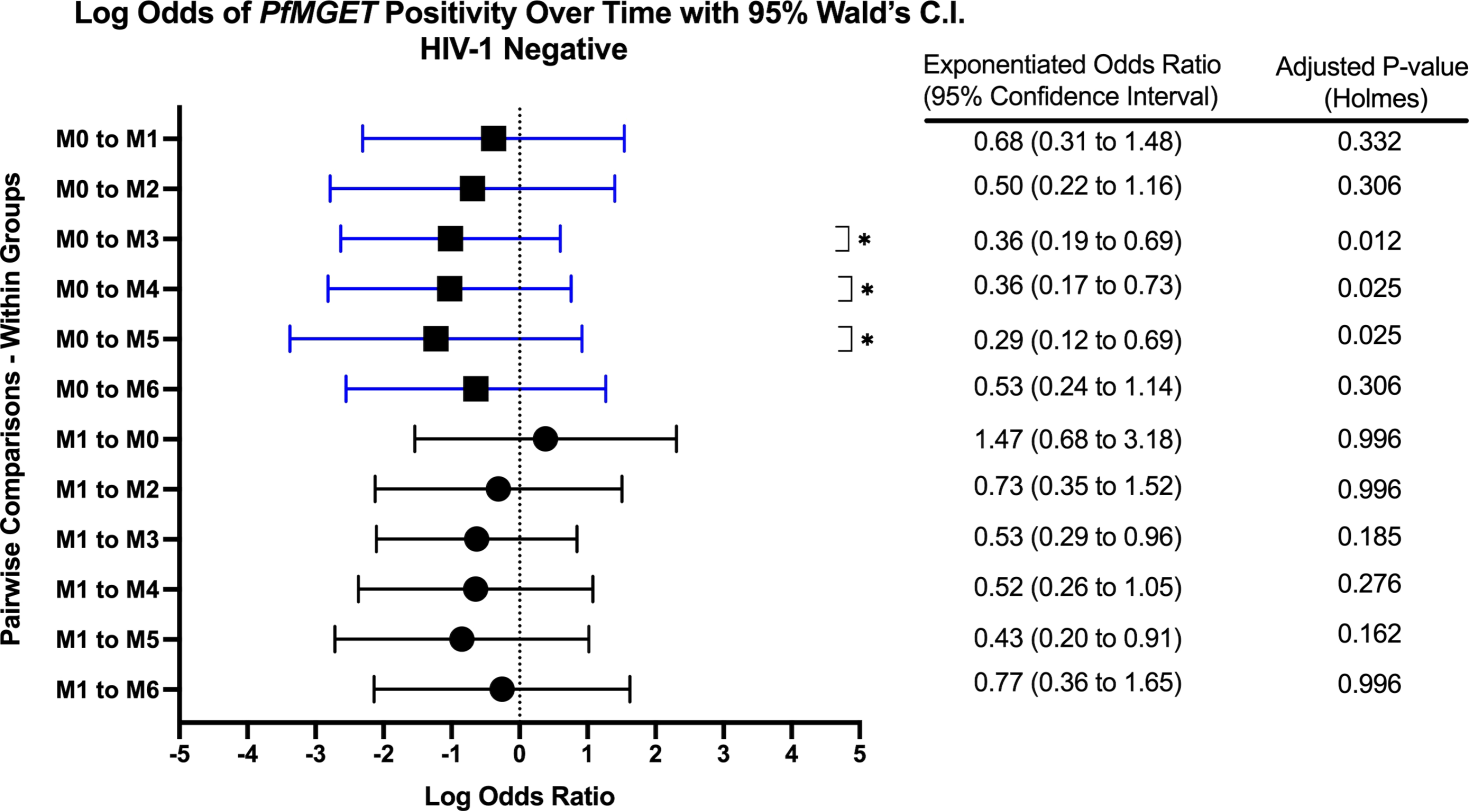
Forest Plot of the Log Odds of *PfMGET* Positivity in HIV-1 Negative Volunteers. The log odds and accompanying exponentiated adjusted odds ratio of being positive for at least one gametocyte specific transcript within the HIV-1 negative study group between month 0 (M0) and between month 1 (M1). P-values were adjusted using the Holm’s method for multiple comparisons. Blue confidence interval lines are comparisons with month 0 and black confidence interval lines are comparisons with month 1.

Within groups over time, there was a significant increase in *PfMGET* positivity for HIV-1 positive volunteers on treatment from month 0 to month 1 (aOR 5.43, adjusted p-value 0.024). HIV-1 positive newly diagnosed volunteers exhibited a tendency for increased positivity at month 5 when compared to month 0 and at month 3 when compared to month 1 (**Figure 7**). HIV-1 negative volunteers exhibited an increase in positivity at months 3, 4, and 5 relative to month 0 (**Figure 8**).

After adjusting for multiple comparisons, *PfMGET* ddPCR concentrations (**Supplemental Figure F**) were not different between groups at each time point or over time within any group. Contrary to the prevalence analysis, the log transformed *18S* copy numbers did not predict *PfMGET* ddPCR concentrations (log transformed). There was no difference in *PfMGET* ddPCR concentrations by gender or age nor any predictive association between WBC, RBC, or CD4+ T-cell levels.

#### 3.5.3 *Pfs16* positive transcripts

Due to low frequencies of *Pfs16* ddPCR positivity, GEE longitudinal analysis was not performed. However, we observed that *Pfs16* positivity trended downward from month 0 to month 1 in HIV-1 positive newly diagnosed volunteers (**Figure 5**).

### 3.6 Some volunteers remained *18S* positive throughout the entire course of study

Eight volunteers (all HIV-1 negative) were *18S* positive at every visit (two lost-to-follow up at month 6). We compared this group of eight volunteers to another group representing all volunteers who were not *18S* positive throughout the entire course of study to determine if they presented with any unique characteristics. While there were some sporadic positive associations with the defined variables (**Supplementary Table 2 and 3**), there was no significant association between any predictor or visit. All eight tested positive for malaria by RDT at some point during the study; therefore, they were treated with AL yet remained positive for asexual parasites.

### 3.7 Association of mosquito infectivity with the prevalence of *18S* or gametocyte-specific transcripts

A total of 184 blood samples used for SMFAs were analyzed for *18S* and all gametocyte markers regardless of *18S* positivity. A total of 34/184 groups of mosquitoes (one group per human blood sample) tested positive for oocysts (at least one mosquito with at least one midgut oocyst). Among the associated human blood samples collected on the day of or within 2 days of mosquito feeding for oocyst-positive SMFA, 50% (17/34) of these blood samples were negative for *18S* and 35% (12/34) were negative for all gametocyte markers. Among the associated human blood samples collected on the day of or within 2 days of mosquito feeding for an oocyst-negative SMFA, 54% were positive for *18S* and 47.7% were positive for at least one gametocyte marker (**Table 2**). By Chi-square analysis, there were no differences in the proportion of *18S* or gametocyte positive samples by study group or oocyst positivity.

**Table 2:** Oocyst Positivity by Study Group and Molecular Diagnostic Positivity.

	Group: Analyzed	*18S* Positive (% of total)	*Pfs25* Positive (% of total)	*PfMGET* Positive (% of total)	*Pfs16* Positive (% of total)	Total
**Oocyst Positive**	HIV (+) ND: 99	7 (38.9%)	9 (50%)	10 (55.6%)	1 (6%)	18
	HIV (-): 85	10 (62.5%)	6 (37.5%)	7 (43.8%)	0 (0%)	16
	**Total**	17 (50%)	15 (44.1%)	17 (50%)	1 (2.9%)	
**Oocyst Negative**	HIV (+) ND: 99	38 (46.9%)	27 (33.3%)	32 (39.5%)	7 (8.6%)	81
	HIV (-): 85	43 (62.3%)	22 (31.9%)	17 (24.6%)	3 (4.3%)	69
	**Total**	81 (54%)	49 (32.7%)	49 (32.7%)	10 (6.7%)	

Number of samples of dissected mosquitoes (~25 per patient blood sample) that had at least one mosquito with at least one midgut oocyst (positive sample) or no mosquitoes with oocysts (negative sample). Each category includes the distribution by study group as well as the respective number of positive samples by molecular marker and their accompanying percentage of the total in each subgroup. HIV (+) ND is HIV-1 positive newly diagnosed volunteers. HIV (-) is HIV-1 negative volunteers.

Among the oocyst positive samples, a range of 12-39 mosquitoes per human blood sample were fed and dissected for oocysts. The mean number of oocysts per mosquito for *18S* positive samples was 1.94 oocysts (range 1-11 oocysts) while the mean for *18S* negative samples was 1.71 oocysts (range 1-7 oocysts) (**Supplementary Table 4**). The mean number of oocysts per mosquito for samples that were gametocyte-positive by ddPCR was 2.14 oocysts (range 1-11 oocysts) while the mean for samples that were gametocyte-negative by ddPCR was 1.25 (range 1-3 oocysts). There was one mosquito pool with a total count of 31 oocysts; this sample was excluded as an outlier and represented a group of mosquitoes fed on a HIV-1 negative sample with detectable parasites by *18S* qPCR and detectable gametocytes by ddPCR. There were no significant differences between oocyst counts by *18S* or gametocyte positivity or study group (unpaired t-test).

In an effort to determine whether any study variables predicted oocyst positivity (successful transmission), selected clinical and diagnostic predictors were analyzed using GEE analysis (**Table 3**). Overall, neither gametocyte positivity nor any individual gametocyte-specific marker were significant predictors of oocyst positivity. Additionally, none of the selected clinical markers were a significant predictor of oocyst positivity.

**Table 3:** GEE Transmission Omnibus Results.

Predictor of Oocyst positivity	Exponentiated aOR	95% Wald Confidence Interval	Adjusted p-value (Holm’s)
Gender (Male : Female)	0.613	0.184 to 2.045	1.000
Study Group (HIV+ ND : HIV-)	1.133	0.409 to 3.140	1.000
Age	0.951	0.906 to 0.998	0.336
CD4+ T cells	1.001	0.999 to 1.002	1.000
HGB	0.975	0.718 to 1.325	1.000
RBC	0.652	0.211 to 2.014	1.000
Gametocyte Positivity (Binary)	1.895	0.842 to 4.264	0.854
*Pfs25* Positivity (Binary)*	1.056	0.403 to 2.766	1.000
*PfMGET* Positivity (Binary)*	1.790	0.626 to 5.119	1.000
*Pfs16* Positivity (Binary)*	0.578	0.090 to 3.697	1.000
Log *18S* Copy numbers/μL	0.984	0.840 to 1.153	1.000

>Predicting overall oocyst positivity using gender, study group, age, CD4+ T cell levels, hemoglobin (HGB) levels (g/L), red blood cell (RBC) counts (x 10^12^/L), binary results for positivity of gametocyte-specific transcripts (positive vs. negative), and log *18S* copy numbers/μL). When log transforming *18S* copy numbers/μL undetermined results were replaced with “0.01” for the transformation. HIV+ ND is HIV-1 positive newly diagnosed volunteers. HIV- is HIV-1 negative volunteers. *Specific gametocyte transcript positivity was analyzed using a separate GEE model than the accompanying predictors.

**Figure 9:**
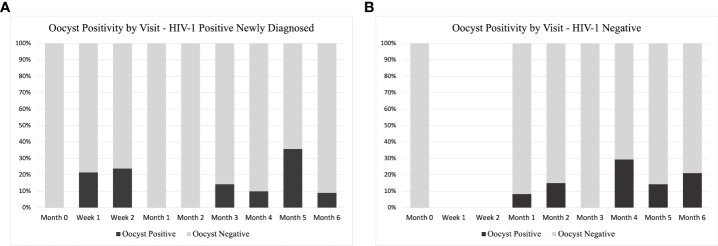
Oocyst Positivity by Study Group and Time. A distribution of the total number of oocyst positive samples as percentage of the total analyzed at each time point for **(A)** HIV-1 positive newly diagnosed volunteers and **(B)** HIV-1 negative volunteers. As part of the study design, HIV-1 negative volunteers did not have a scheduled visit at week 1 or week 2, so no samples were analyzed at these time points.

Due to lack of data at certain time points (**Figure 9B**), we were unable to compare oocyst positivity over time. However, for HIV-1 positive newly diagnosed volunteers, week 1 and week 2 account for 44.4% of all oocyst positivity. There were no oocyst positive samples in the subsequent month 1 and month 2.

### 3.8 RDT positivity at baseline was positively associated with gametocyte prevalence at baseline for HIV-1 positive newly diagnosed volunteers

If a participant was HIV-1 positive newly diagnosed and RDT positive at baseline (month 0), they had a significantly higher probably of also being positive for at least one gametocyte specific transcript when compared to HIV-1 negative volunteers and HIV-1 positive volunteers on treatment (Chi-square test *p-*values 0.005 and 0.001, respectively) (**Table 4**).

**Table 4:** Comparison of Gametocyte Positivity at Month 0 by RDT positivity and Study Group.

Study Group		RDT Negative (% of Total)	RDT Positive (% of Total)	Total
HIV (-)	Negative for Gametocytes	26 (68.4%)	12 (31.6%)	38
	Positive for Gametocytes	20 (64.5%)	11 (35.5%)	31
HIV (+) ND	Negative for Gametocytes	19 (70.4%)	8 (29.6%)	27
	Positive for Gametocytes	4 (20.0%)	16 (80.0%)	20
HIV (+) OT	Negative for Gametocytes	13 (100%)	0 (0.0%)	13
	Positive for Gametocytes	11 (84.6%)	2 (15.4%)	13

A comparison by study group of the interaction between gametocyte positivity and RDT positivity at baseline (month 0). Numbers of positive or negative samples and the percentages (in paratheses) of the total number analyzed are provided. HIV (-) is HIV-1 negative volunteers. HIV (+) ND is HIV-1 positive newly diagnosed volunteers. HIV (+) OT is HIV-1 positive volunteers on treatment for ART and TS.

### 3.9 Impact of ART and antimalarials on gametocyte positivity after treatment

**Figure 10:**
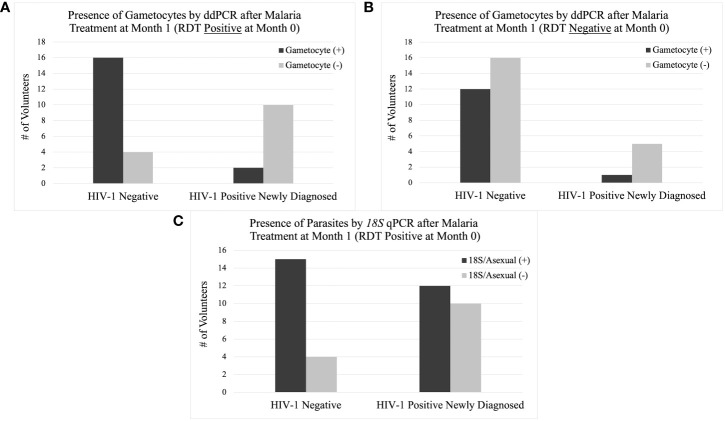
Presence of Gametocytes and Asexual Parasites at Month 1 by Study Group. The column graph on the top left **(A)** shows the presence of gametocytes by ddPCR after AL treatment at month 1 if the volunteer was positive for malaria by RDT at month 0. The column graph on the top right **(B)** shows the presence of gametocytes by ddPCR without AL treatment at month 1 if the volunteer was negative for malaria by RDT at month 0. The column graph on the bottom center **(C)** shows the presence of asexual parasites by *18S* qPCR after AL treatment at month 1 if the volunteer was positive for malaria by RDT at month 0.

If a volunteer was positive for malaria by RDT at any time, they were treated with AL in accordance with Kenyan MoH guidelines. Among the 782 samples that were subsequently tested for gametocytes (*18S* positive), HIV-1 positive newly diagnosed volunteers had a significantly lower probability of being gametocyte positive in the subsequent month (month 1) after initiation of ART, TS, and AL at month 0 (RDT positive) compared to HIV-1 negative volunteers after initiation of just AL at month 0 (**Table 5** and **Figures 10A, B**). The difference was significant using Pearson’s Chi-square (*p-*value: 0.003) despite there being no statistical difference in asexual parasite clearance (*18S* positivity) (*p*-value: 0.1892) (**Table 5** and **Figure 10C**). Comparisons between HIV-1 volunteers on treatment were not conducted because only two samples were positive by RDT at month 0.

**Table 5:** Comparison of Gametocyte and *18S* Positivity at Month 0.

	Study Group	Positive for Gametocytes (% within group)	Negative for Gametocytes (% within group)	Total
**RDT Positive Month 0**	HIV (-)	11 (73.3%)	4 (26.7%)	15
	HIV (+) ND	2 (16.7%)	10 (83.3%)	12
	Total	13	14	27
RDT Positive	Study Group	Positive for Gametocytes (% within group)	Negative for Gametocytes (% within group)	Total
**Month 0**
HIV (-)	12 (42.9%)	16 (57.1%)	28
HIV (+) ND	1 (16.7%)	5 (83.3%)	6
Total	13	21	34
RDT Positive	Study Group	Positive for Gametocytes (% within group)	Negative for Gametocytes (% within group)	Total
**Month 0**
HIV (-)	15 (78.9%)	4 (21.1%)	19
HIV (+) ND	12 (54.6%)	10 (45.4%)	22
Total	27	14	41

Gametocyte prevalence by ddPCR after malaria treatment at month 1 if the volunteer was positive by RDT at month 0. Gametocyte prevalence by ddPCR in the absence of malaria treatment at month 1 if the volunteer was negative by RDT at month 0. *18S* prevalence by qPCR after malaria treatment at month 1 if the volunteer was positive by RDT at month 0.

This trend was only observed from month 0 to month 1 when comparing gametocyte positivity in the subsequent month after testing positive for malaria by RDT. There were no other time points that showed a significant difference in gametocyte positivity after prescribed antimalarial treatment.

In an effort to determine which intervention (ART and TS or AL) was associated with the change in gametocyte prevalence, we compared the same groups that were malaria RDT positive at month 0 to those that were malaria RDT negative at month 0. For samples that were RDT negative at month 0, the significant difference in gametocyte prevalence at month 1, observed in RDT positive samples, disappeared between the HIV-1 newly diagnosed volunteers and the HIV-1 negative volunteers (*p-*value 0.231) (**Table 5** and **Figures 10A, B**). HIV-1 negative volunteers who did not receive AL (RDT negative) exhibited a decrease in the percentage of individuals who were positive for gametocytes at month 1 (**Table 5** and **Figure 10B**). Neither group received AL treatment; however, HIV-1 positive newly diagnosed volunteers still received ART and TS. Using a proportions test to compare the two different RDT groups (AL vs. no AL), there was a significant difference in the ratio of gametocyte positivity at month 1 for the HIV-1 negative volunteers (*p-*value 0.023) while HIV-1 positive newly diagnosed volunteers maintained a similar difference in the ratio of gametocyte positivity between the two categories (*p-*value 1.000).

## 4 Discussion

This study investigated the longitudinal impact of HIV-1 co-infection and drug treatment on gametocyte transcript prevalence and parasite transmission to *A. gambiae* from asymptomatic volunteers in a region of holoendemic malaria transmission. The overall prevalence for at least one gametocyte specific transcript in *18S* positive individuals across all groups and at all time points was 51.1% (400/782) using ddPCR assays. The GEE models showed that there was no significant effect on, or interaction between, the HIV-1 status and/or the visit number (time) and gametocyte positivity. However, as expected, there was a significant relationship between the log transformed *18S* copy numbers and gametocyte transcript prevalence (aOR: 1.202, adjusted *p-*value 0.018). The odds of being gametocyte positive varied by HIV status and time point, but there was no significant predictive interaction. There was a tendency for increased *Pfs25* and *PfMGET* transcript prevalence after month 0 and/or month 1 (**Figures 5–8**). For HIV-1 positive newly diagnosed volunteers, the initiation of ART and TS at month 0 (despite receiving antimalarials if RDT positive) was associated with a significant impact on the reduction of gametocyte transcript prevalence in the subsequent month (**Figure 10A**). Interestingly, 50% of the blood samples that were associated with mosquito infection by oocysts were negative for malaria by *18S* qPCR and 35% were negative for all gametocyte specific transcript markers by ddPCR. There were no significant differences in HIV-1 status, time, or gametocyte prevalence and oocyst positivity.

In areas of holoendemic malaria transmission, such as the study area near Kisumu, Kenya, clinical immunity to malaria develops during childhood and continues into adulthood due to the frequency of new infections (Djimde et al., 2003; Rogerson et al., 2010). The high density of semi-immune asymptomatic individuals likely serves as a primary reservoir for malaria parasite transmission. Among the 300 malaria asymptomatic volunteers enrolled in the study, 263 (87.7%) were positive for malaria parasites by *18S* qPCR at least once throughout the 6-month course of the study and 73% of those malaria positive volunteers were positive for gametocyte specific transcripts at least once by ddPCR. With this longitudinal study design, we could include patients that shifted from detectable to undetectable parasitemias and gametocytemias, a cyclic commonality well described for malaria parasites (Bruce and Day, 2002). When compared to the baseline point prevalence (47.7% parasite prevalence and 45.5% gametocyte prevalence) (**Figure 1**), the inclusion of multiple time points increased the ability to detect parasites in individuals by 84 percent and to detect gametocytes in individuals by 60 percent. The high positivity seen throughout the study reflect the endemicity of asymptomatic parasitemias and the fact that semi-immune individuals, regardless of HIV-1 status, cycle between detectable and undetectable parasitemias and gametocytemias. Eight HIV-1 negative volunteers were positive for malaria parasites by *18S* qPCR every month over the 6-month study period; however, they were not positive for gametocytes at every time point. From this study, we were unable to identify any unique predictors about these individuals and why they maintained chronic parasitemias despite prescribed AL treatment.

HIV-1 and malaria co-infection has been associated with poor clinical outcomes (Kublin et al., 2005; Hewitt et al., 2006; Kiyingi et al., 2010; Flateau et al., 2011), but little is known about the impact of asymptomatic co-infection on malaria parasite transmission potential. In our previous point-prevalence study of parasites and gametocytes in asymptomatic individuals at the time of HIV-1 testing (n=1,116), the relative risk for gametocyte positivity was 1.82 higher for HIV-1 positive samples than HIV-1 negative samples (Stiffler et al., 2020). However, when evaluating the point prevalence at baseline (n=143) of our current study, we did not see the same trend. In fact, gametocyte positivity was nearly equal between HIV-1 negative and HIV-1 positive (32/70 and 33/73 respectively) (**Figure 1**). It is likely that the sample size in this longitudinal study was not large enough to replicate the same results.

An important aim of our longitudinal study was to evaluate the impact of ART and TS treatment on asymptomatic gametocytemias over an extended period of time. The antifolate drugs TS and sulfadoxine-pyrimethamine (SP) share pharmacological similarities and SP resistance is well described in the geographical area of our study (Barnes et al., 2008; Oesterholt et al., 2009). TS has been described to have an inhibitory effect on gametocyte burden and parasite transmission in *in vitro* models (Hobbs et al., 2012; Hobbs et al., 2013). Another study demonstrated that antifolate therapy enhances gametocytemia almost immediately after drug initiation and peaks at about two weeks into therapy (Bousema et al., 2006), but there was only one month of follow-up in that study. Our longitudinal study design included visits for HIV-1 newly diagnosed volunteers at one week and two weeks post initiation of ART and TS. Within those two weeks, there was an observed increase in *Pfs25* and *PfMGET* prevalence with a decrease in *Pfs16* prevalence (**Figure 4A**). Unfortunately, due to the study design, week 1 and week 2 data were not collected on HIV-1 negative volunteers to use as a reference for statistical analysis and, therefore, the HIV-1 positive newly diagnosed data for these time points was not included. However, throughout the course of our longitudinal study we did not observe any significant reduction in gametocyte transcript prevalence or ddPCR concentration that could be associated with ART or TS treatment.

Based on GEE analysis to compare results from month 0 to month 6, HIV-1 positive newly diagnosed volunteers exhibited a tendency for increased gametocyte positivity after month 1 and a drop in positivity at month 4. HIV-1 negative volunteers also exhibited a moderate tendency for increased gametocyte positivity over time with an inexplicable drop in positivity at month 5 (**Figure 5B**). A tendency for early increased gametocyte positivity after antifolate treatment was reported by Bousema et al. (Bousema et al., 2006), but the increases we observed were independent of HIV status and treatment. The unexplained drops were not a result of seasonality as the drops fell on different calendar months (**Supplemental Figure G**), suggesting a biological pattern that deserves further study. There were significant differences among groups, but there were no differences between groups over time. Given that only 42% of the 300 enrolled volunteers presented at every visit/time point which limited our analyses, the lack of significance could have resulted from high malaria prevalence and sample sizes that were too small to detect differences in positivity.

Within the HIV-1 positive newly diagnosed group, we observed a steep increase in *PfMGET* positivity between enrollment (month 0) to week 2 (8% to 31%) (**Figure 4A**). While positivity by the gametocyte specific transcript marker *PfMGET* is not a direct indicator of the quantity of male gametocytes present, it does indicate that at least one male gametocyte was present in the dried blood spot. Changes in quantity of male gametocytes have been associated with anemia, parasitic competition, and changes in immunity [reviewed in (Roberds et al., 2021)]. Anemia (RBC and/or hemoglobin levels) was analyzed in this study, but there were no significant associations between *PfMGET* positivity or any other gametocyte specific marker with RBC or hemoglobin levels. Parasites are believed to alter their sex ratios with an increase in male gametocytes as a form of fertility insurance (Reece et al., 2009; Ramiro et al., 2011). This prediction also suggests that transmission blocking immunity (TBI) might drive an increase in male gametocyte production. While further study is required to test mechanism, it is possible the first two weeks of ART and changes to host immunity contribute to increased positivity of male specific transcripts to a level that was equal to that of female specific transcript positivity.

We also examined associations with antimalarial treatment in the subsequent month after treatment. Compared to HIV-1 negative volunteers, there was a significant difference in gametocyte positivity at month 1 for the HIV-1 newly diagnosed volunteers who were RDT positive at month 0 and prescribed AL (*p-*value 0.003) (**Table 5** and **Figure 10A**). Interestingly, a significantly lower proportion of samples from HIV-1 positive newly diagnosed volunteers were gametocyte positive Comparing the groups at the same time point but without antimalarial treatment, gametocyte prevalence outcome for HIV-1 positive newly diagnosed volunteers stayed the same while the outcome for HIV-1 negative volunteers changed. In samples without AL treatment at month 0, there was a significantly higher likelihood for lower gametocyte positivity than if they were treated (*p-*value 0.023). These observations suggest that the combination of ART and TS with antimalarials could be associated with increased clearance of gametocytes compared to AL treatment alone, which was associated with increased gametocyte prevalence.

Gametocyte prevalence is often described to have an association with transmission potential, simply because gametocyte maturation is the precursor required for parasite fertilization and oocyst development in the mosquito. However, transmission potential and success should not be defined without the context of infectivity to the mosquito through oocyst or sporozoite enumeration (Muirhead-Thomson, 1954). This concept remains true regardless of improved methods for detection of gametocytes. There were no significant predictors of oocyst positivity or oocyst enumeration, including HIV-1 status, log transformed *18S* copy numbers/μL or presence or concentration of common gametocyte specific markers. In fact, only 65% of mosquito groups considered to be oocyst positive were associated with gametocyte-positive volunteer blood samples collected on the day of or within two days of SMFA. Further, half of all blood samples associated with oocyst-positive mosquito groups failed to test positive for parasites by *18S* qPCR (**Table 2**). It is well known that submicroscopic infections can successfully transmit to mosquitoes [reviewed in (Nassir et al., 2005; Babiker et al., 2008)], but our data show that transmission can also occur at densities that are below detection of highly sensitive molecular assays. This underscores recent findings from a controlled human malaria infection that show infectivity occurred at rates as low as 1.6 gametocytes/μL of blood (5 million RBC) (Collins et al., 2018), and confirms that actual mosquito infectivity remains the only accurate measurement of transmission.

Increased *PfMGET* positivity from enrollment (month 0) to week 2 for HIV-1 newly diagnosed volunteers was associated with 44% of all oocyst-positive mosquito groups fed on volunteer blood in weeks 1 and 2 from this group (**Figure 9A**). Despite a lack of data from the HIV-1 negative control group for comparison, these observations suggest that analyses of the effects of ART and TS on parasite sex ratio and potential for transmission success to mosquitoes might be warranted. Altered sex ratio could drive diversity of infecting drug resistant genotypes. Competition and inbreeding can increase male gametocyte production (Reece et al., 2008), so drug resistant parasite genotypes might infect mosquitoes more efficiently (Méndez et al., 2007; Mharakurwa et al., 2011).

The use of ddPCR, which is more sensitive than microscopy or qPCR (Taylor et al., 2017), requires multiple calculations and assumptions to determine absolutely copy number concentration that can be a limitation of this technology (Koepfli et al., 2016; Taylor et al., 2017). The use of plasmid standards to compare concentrations versus copies/μL yielded agreement, but the concentrations were always smaller than the equivalent copies/μL determined by standards in qPCR. Many of the samples analyzed were positive for gametocyte specific transcripts but at very low ddPCR concentrations that made it difficult to compare concentrations between samples. Additionally, ddPCR assays require a user to set a threshold to determine the cutoff amplitude at which a droplet is either positive or negative. The separation between positive and negative droplets specifically within the *PfMGET* marker included “rain” droplets that were difficult to classify as positive or negative. Rain droplets can be due to false positive readings, an inhibited or “lagging” PCR reaction, coagulation of multiple droplets, or primer-target mismatch (Strain et al., 2013; Trypsteen et al., 2015). To design a non-biased approach for setting the threshold for the *Pfs25* and *PfMGET* duplex assay, we used a conservative method to determine concentrations (ddpcRquant) (Trypsteen et al., 2015). In optimizing the *Pfs16* marker for ddPCR, we observed more distinct separation between positive and negative droplets (rain droplets were not common) which allowed a manual threshold to be set with minimal bias.

While we did not observe any significant associations with predicted clinical factors and HIV-1 status in this longitudinal study, our data highlight some of the complexities surrounding HIV-1 malaria co-infection and transmission potential to mosquito vectors. We suggest that future studies should focus on the impact that ART, antimalarials, and antifolate drugs have on gametocyte burden and subsequent mosquito infectivity using more frequent visits over time. Additionally, we recommend that future studies should closely monitor changes in parasite genotypes and chronicity of infection over a longer study duration to better track changes in CD4+ T-cell counts. From a global health perspective, our findings highlight the ongoing need to focus on areas with high asymptomatic infection rates and complex transmission patterns and to support malaria elimination strategies.

## Data availability statement

All relevant data is contained within the article: The original contributions presented in the study are included in the article/**Supplementary Material**, further inquiries can be directed to the corresponding author/s.

## Ethics statement

Ethical approval for human use and supervision of ethical sample collection was granted and managed by the Walter Reed Institute of Research (WRAIR) Institutional Review Board (WRAIR #2346) and the Scientific and Ethics Review Unit of the Kenya Medical Research Institute (SERU-KEMRI, SSC #3606).

## Author contributions

SL and VS conceived and designed the longitudinal study. AR designed the ddPCR gametocyte assays with guidance from ZL. JO and CK contributed to human sample collection and analysis in Kenya. JM, DO, and CW contributed to mosquito SMFAs, dissections, and analysis in Kenya. AR and CK contributed to data collection. AR performed statistical analysis and drafted the manuscript. All authors contributed to the article and approved the submitted version.

## Funding

This work was supported by NIH NIAID R01 AI104423 (VS, SL).

## Acknowledgments

The authors would like to thank the study participants and clinical staff for their involvement in this work. We would also like to thank Dr. Cara Olsen and Ms. Sorana Raiciulescu and (Uniformed Services University of the Health Sciences) for their expertise and advice on statistical analyses. We thank Mr. Andrew Frank (Student Bioinformatics Initiative at the Uniformed Services University of Health Sciences) for helpful discussions and providing high-performance computing resources. Additionally, we would like to thank Mr. Truong Luu, Mr. Nathaniel Dizon, and Ms. Karina Rivas (Uniformed Services University of the Health Sciences) for their technical assistance.

## Conflict of interest

The authors declare that the research was conducted in the absence of any commercial or financial relationships that could be construed as a potential conflict of interest.

## Publisher’s note

All claims expressed in this article are solely those of the authors and do not necessarily represent those of their affiliated organizations, or those of the publisher, the editors and the reviewers. Any product that may be evaluated in this article, or claim that may be made by its manufacturer, is not guaranteed or endorsed by the publisher.

## Author disclaimer

The opinions and assertions expressed herein are those of the author(s) and do not reflect the official policy or position of the Uniformed Services University of the Health Sciences or the Department of Defense.

References to non-Federal entities or products do not constitute or imply a Department of Defense or Uniformed Services University of the Health Sciences endorsement.
