# Supplementary material for: Longitudinal impact of asymptomatic malaria/HIV-1 co-infection on Plasmodium falciparum gametocyte transcript expression and transmission to Anopheles mosquitoes
Source: Front Cell Infect Microbiol. 2022 Sep 16;12:934641. doi: 10.3389/fcimb.2022.934641 (PMC9523792; doi:10.3389/fcimb.2022.934641)
Supplement: Supplementary file 1 [file DataSheet_1.docx]

Supplementary Material

## Supplementary Figures


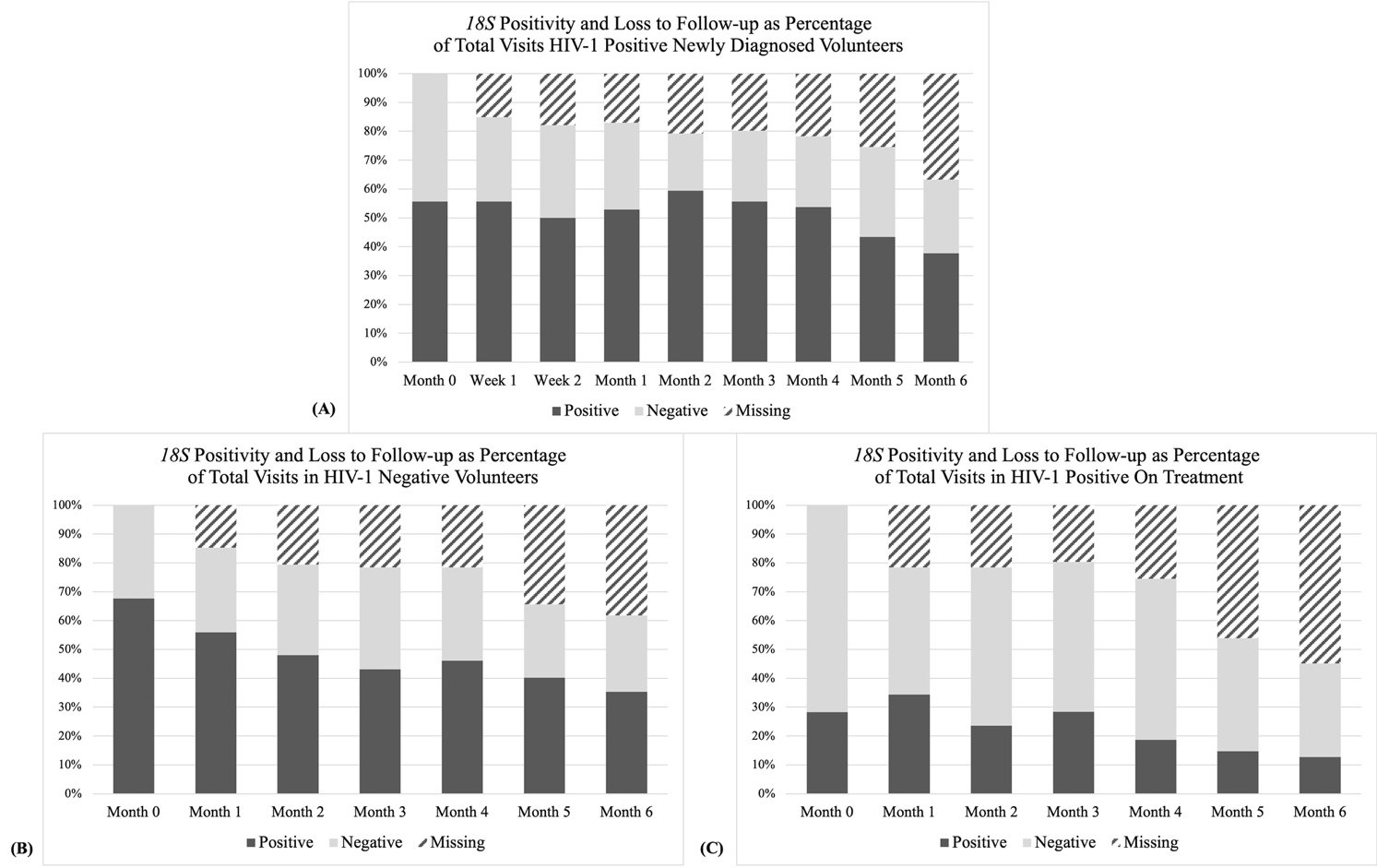


**Supplementary Figure A.** ***18S* Positivity Over Time and Study Group**. The percentage of *18S* positive, *18S* negative, and missing samples (due to loss-to-follow up) for each study group over the course of the 6-month study. **(A)** HIV-1 positive newly diagnosed volunteers **(B)** HIV-1 positive volunteers on treatment **(C)** HIV-1 negative volunteers.


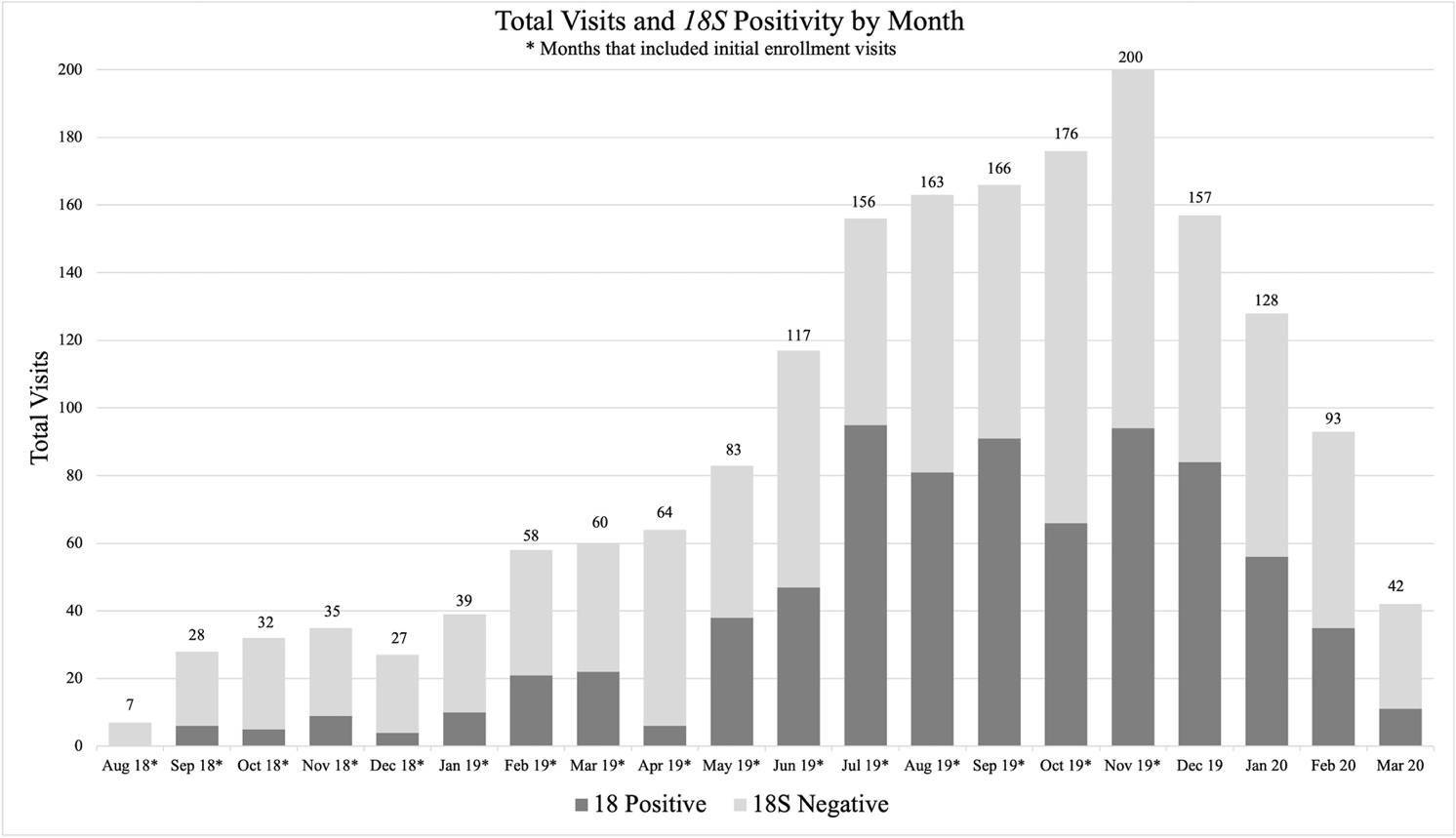


**Supplementary Figure B.** **Distribution of *18S* Positive and Negative Samples by Calendar Month.**

**
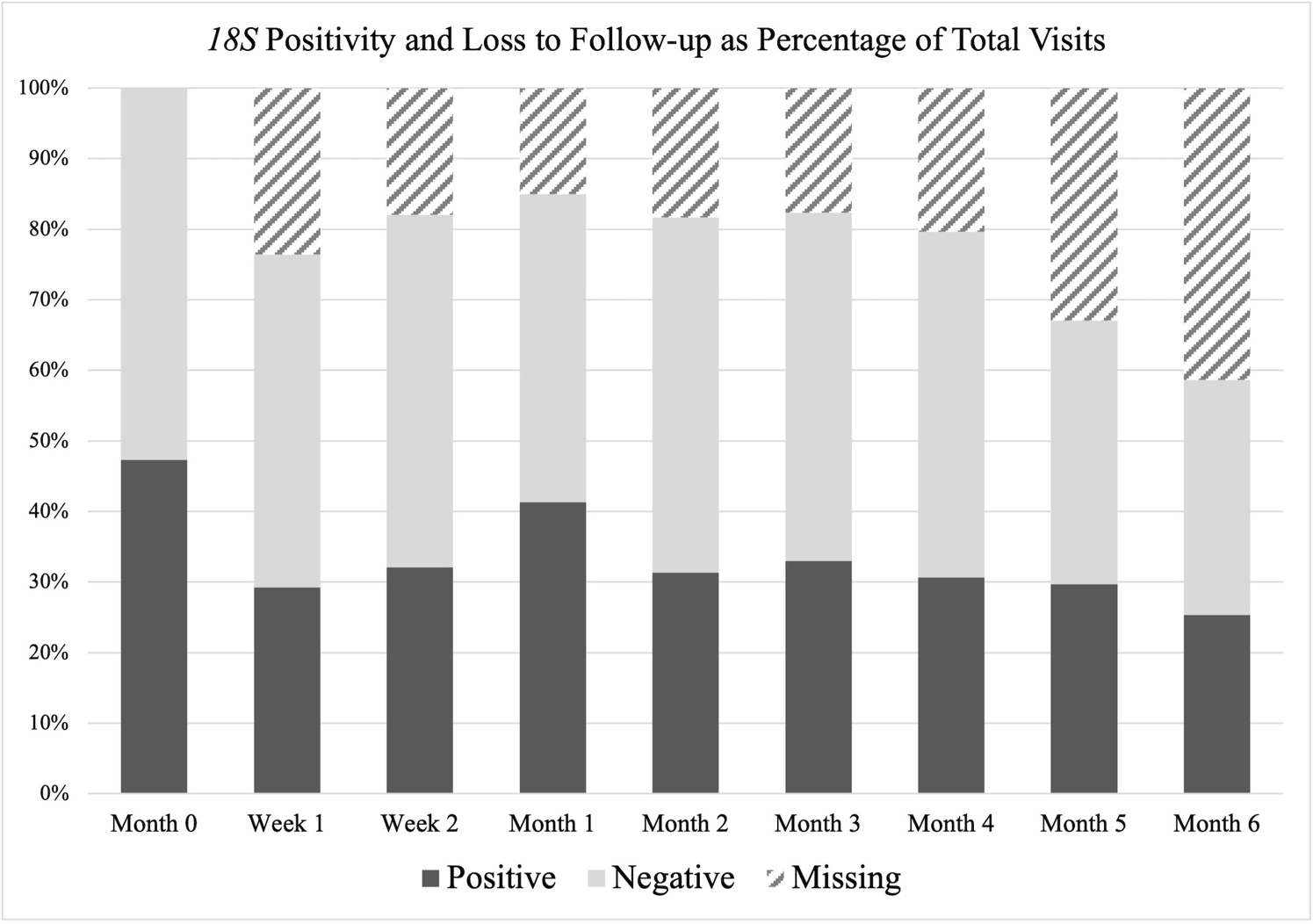
**

**Supplementary Figure C. *18S* Positivity Overtime for all Study Groups**. The percentage of *18S* positive, *18S* negative, and missing samples (due to loss-to-follow up) for all study groups combined over the course of the 6-month study.

**
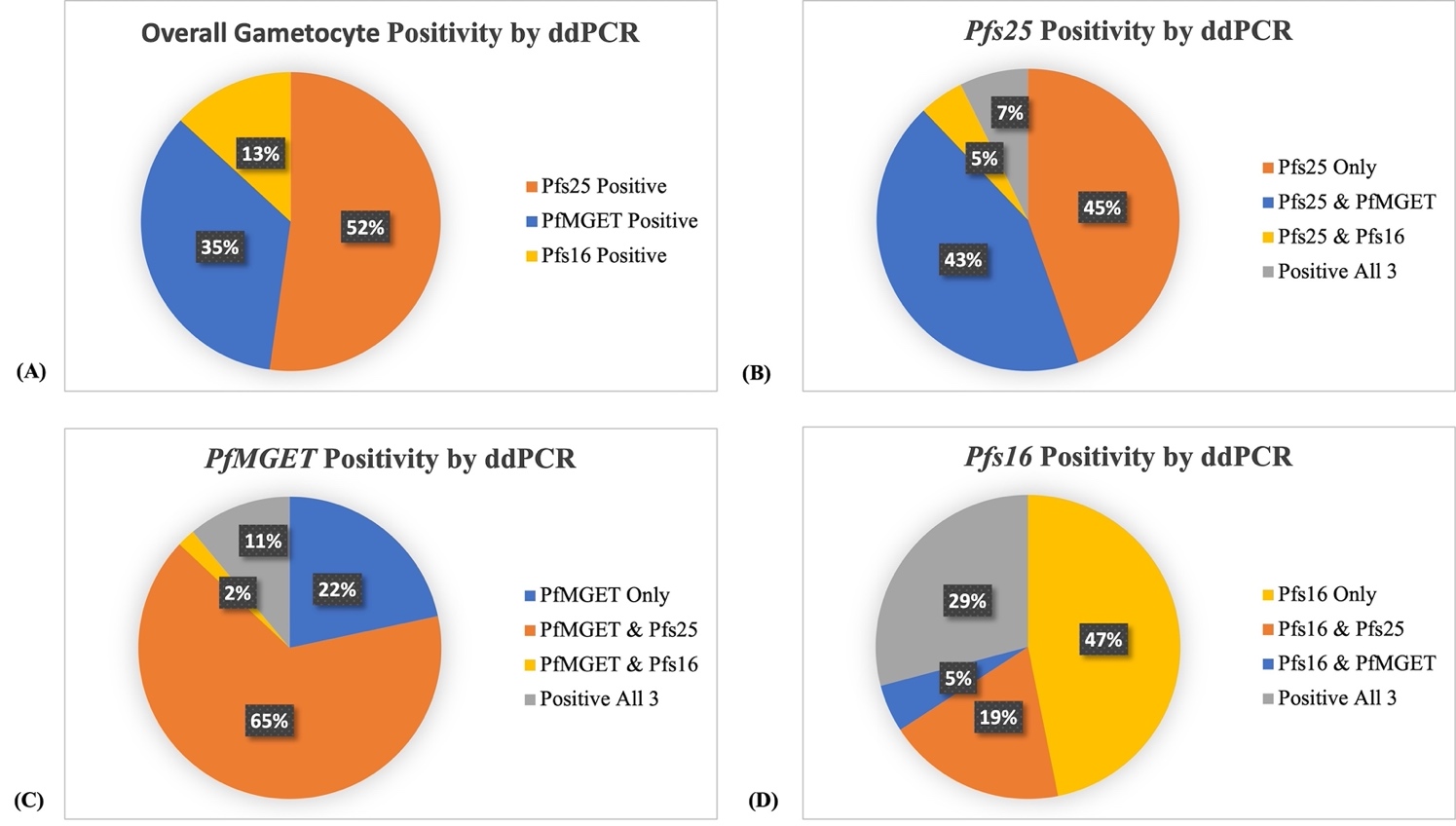
**

**Supplementary Figure D. Percent Positivity by Gametocyte-Specific ddPCR Molecular Marker. (A)** Overall gametocyte positivity by ddPCR **(B)** *Pfs25* positivity by ddPCR **(C)** *PfMGET* positivity by ddPCR and **(D)** *Pfs16* positivity by ddPCR.


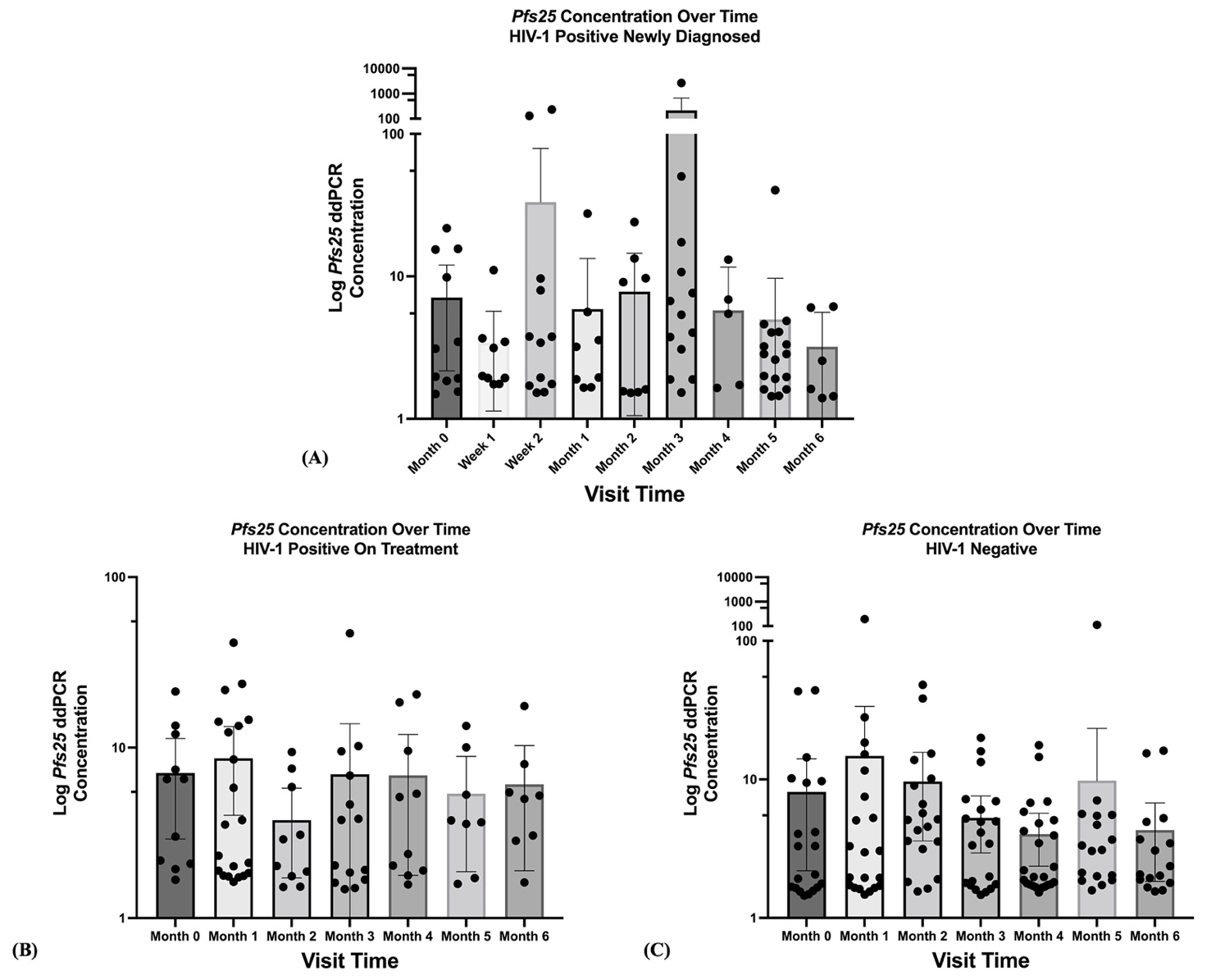


**Supplementary Figure E. ddPCR Concentration of *Pfs2*5 by Study Group Over Time**. **(A)** HIV-1 positive newly diagnosed volunteers **(B)** HIV-1 positive volunteers on treatment **(C)** HIV-1 negative volunteers. Includes the mean bar and 95% confidence interval (above Log 1).


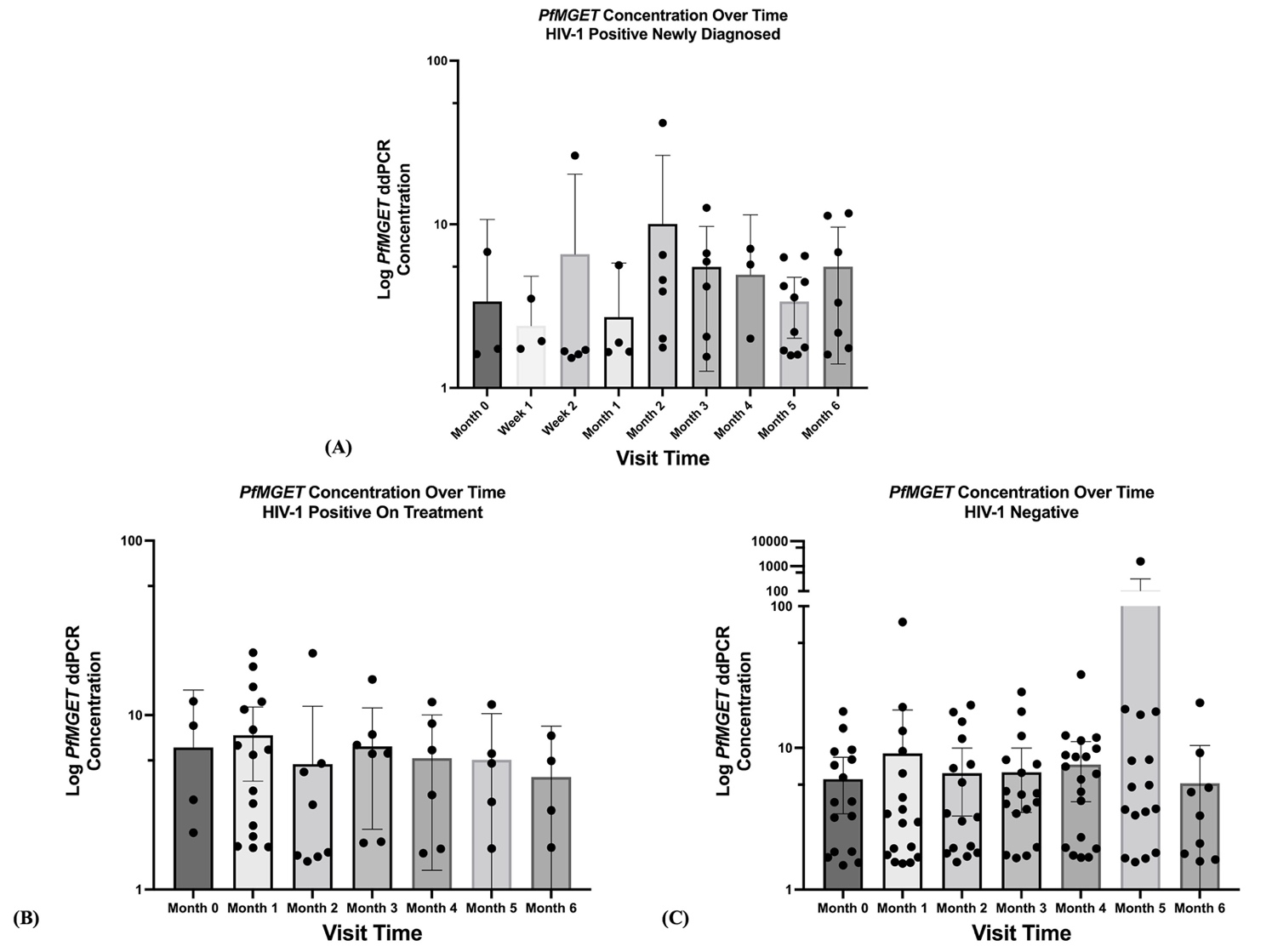


**Supplementary Figure F.** **ddPCR Concentration of *PfMGET* by Study Group Over Time**. **(A)** HIV-1 positive newly diagnosed volunteers **(B)** HIV-1 positive volunteers on treatment **(C)** HIV-1 negative volunteers. Includes the mean bar and 95% confidence interval (above Log 1).


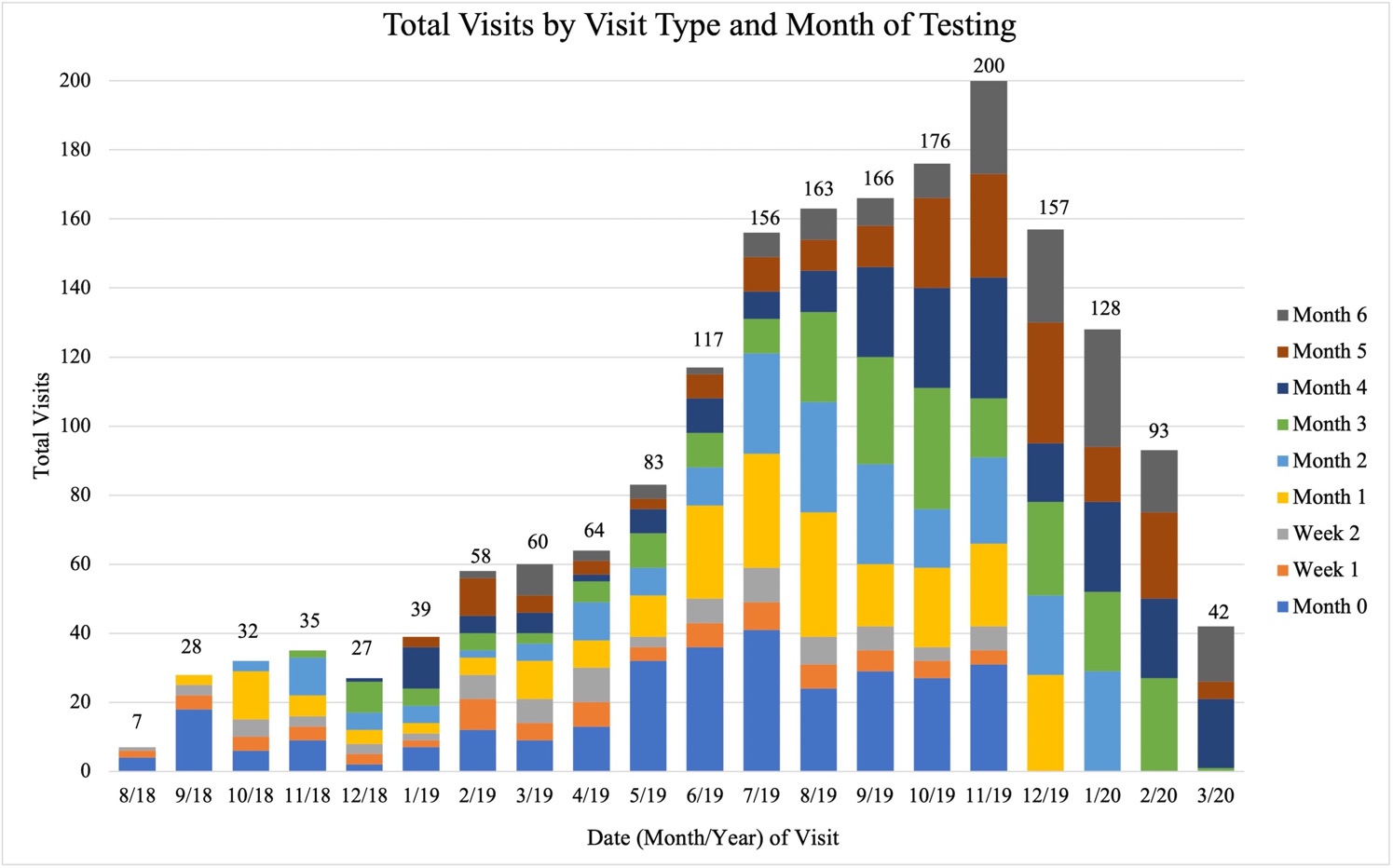


**Supplementary Figure G. Study Visit by Calendar Month/Year.**

## Supplementary Tables

**Supplementary Table 1.** ddPCR primers (Integrated DNA Technologies, Coralville, IA, US) and probe (Sigma-Aldrich, St. Louis, MO, US) sequences. The 5’ end of each probe is labeled with a fluorophore (FAM or HEX). The 3’ end of each probe is labeled with a black hole quencher (BHQ-1).

| Target | Forward Primer, Reverse Primer, and Probe Sequences | | Adapted from: |
| --- | --- | --- | --- |
| *pfs25*  (Pf3D7_1031000) | **Forward** | 5' TCTGAAATGTGACGAAAAGACTGT 3' | (Stiffler et al., 2020) |
|  | **Reverse** | 5' AGCGTATGAAACGGGATTTCC 3' |  |
|  | **Probe** | FAM 5’ ATAAACCATGTGGAGATTT 3’ BHQ-1 |  |
| *pfMGET*  (Pf3D7_1469900) | **Forward** | 5’ AAAATTCGGTCCAAATATAAAATCCTG 3’ | (Wang et al., 2020) |
|  | **Reverse** | 5’ CTTCATCAATTAAAAATCCCTTTTTTGT 3’ |  |
|  | **Probe** | HEX 5’ CCTGGTAAAAAACAGCTCCAGCA 3’ BHQ-1 |  |
| *pfs16*  (Pf3D7_0406200) | **Forward** | 5’ GGATCCCCTTCAACTTTGCA 3’ | (Stiffler et al., 2020) |
|  | **Reverse** | 5’ CCTTGAGATAGTCCACCTTGATTAGG 3’ |  |
|  | **Probe** | FAM 5’ TTCTTCAGGTGCCTCTCTTCATGCTGTTG 3’ BHQ-1 |  |

**Supplementary Table 2.** P-value chart comparing eight individuals who were *18S* positive the entire study to all other individuals in the study who were not *18S* positive the entire study (Mann-Whitney nonparametric test). Green highlighted cells indicate significance (p-values) less than 0.05. Predictors include *18S* copy numbers/μL, *Pfs25, PfMGET,* and *Pfs16* concentrations (Conc.), CD4+ T cell levels, white blood cell (WBC) counts (x 10^9^/L), red blood cell (RBC) counts (x 10^12^/L), hemoglobin (HGB) levels (g/L), neutrophils (NEU) (%), lymphocytes (LYM) (%), monocytes (MON) (%), platelets (PLT) (fL), and age.

|  | ***18S*  Copy #/uL** | ***Pfs25***  **Conc.** | ***PfMGET***  **Conc.** | | ***Pfs16***  **Conc.** | **CD4+**  **T cells** | | **WBC** | | **RBC** | | **HGB** | | **NEU** | | **LYM** | | **MON** | | **PLT** | | **Age** |  |
| --- | --- | --- | --- | --- | --- | --- | --- | --- | --- | --- | --- | --- | --- | --- | --- | --- | --- | --- | --- | --- | --- | --- | --- |
| **Month 0** | 0.097 | 0.861 | 0.528 | 0.872 | | 0.200 | 0.626 | | 0.116 | | 0.815 | | 0.190 | | 0.068 | | 0.184 | | 0.333 | | 0.177 | | |
| **Month 1** | 0.011 | 0.873 | 0.920 | 0.416 | | 0.025 | 0.835 | | 0.242 | | 0.827 | | 0.416 | | 0.847 | | 0.063 | | 0.051 | |  | | |
| **Month 2** | 0.035 | 0.580 | 0.049 | 0.613 | | 0.230 | 0.664 | | 0.401 | | 0.802 | | 0.140 | | 0.494 | | 0.834 | | 0.468 | |  | | |
| **Month 3** | 0.070 | 0.592 | 0.625 | 0.255 | | 0.125 | 0.275 | | 0.068 | | 0.653 | | 0.386 | | 0.106 | | 0.939 | | 0.110 | |  | | |
| **Month 4** | 0.026 | 0.492 | 0.719 | 0.396 | | 0.058 | 0.043 | | 0.080 | | 0.779 | | 0.212 | | 0.420 | | 0.584 | | 0.100 | |  | | |
| **Month 5** | 0.002 | 0.475 | 0.833 | 0.270 | | 0.180 | 0.585 | | 0.027 | | 0.305 | | 0.971 | | 0.758 | | 0.045 | | 0.178 | |  | | |
| **Month 6** | 0.068 | 0.201 | 0.616 | 0.128 | | 0.101 | 0.328 | | 0.233 | | 0.646 | | 0.412 | | 0.266 | | 0.029 | | 0.049 | |  | | |

**Supplementary Table 3**. P-value chart comparing eight individuals who were *18S* positive the entire study to all other HIV-1 negative individuals in the study who were not *18S* positive the entire study (Mann-Whitney nonparametric test). Green highlighted cells indicate significance (p-values) less than 0.05. Predictors include *18S* copy numbers/μL, *Pfs25, PfMGET,* and *Pfs16* concentrations (Conc.), CD4+ T cell levels, white blood cell (WBC) counts (x 10^9^/L), red blood cell (RBC) counts (x 10^12^/L), hemoglobin (HGB) levels (g/L), neutrophils (NEU) (%), lymphocytes (LYM) (%), monocytes (MON) (%), platelets (PLT) (fL), and age.

|  | ***18S*  Copy #/uL** | ***Pfs25***  **Conc.** | ***PfMGET***  **Conc.** | ***Pfs16***  **Conc.** | **CD4+**  **T cells** | **WBC** | **RBC** | **HGB** | **NEU** | **LYM** | **MON** | **PLT** | | | **Age** |
| --- | --- | --- | --- | --- | --- | --- | --- | --- | --- | --- | --- | --- | --- | --- | --- |
| **Month 0** | 0.150 | 0.926 | 0.960 | 0.482 | 0.378 | 0.248 | 0.249 | 0.881 | 0.157 | 0.139 | 0.080 | 0.344 | | | 0.733 |
| **Month 1** | 0.520 | 0.710 | 0.932 | 0.301 | 0.863 | 0.558 | 0.435 | 0.936 | 0.469 | 0.945 | 0.040 | 0.141 | | |  |
| **Month 2** | 0.176 | 0.538 | 0.038 | 0.962 | 0.126 | 0.588 | 0.892 | 0.989 | 0.234 | 0.379 | 0.291 | 0.588 | | |  |
| **Month 3** | 0.523 | 0.430 | 0.972 | 0.148 | 0.241 | 0.044 | 0.301 | 0.772 | 0.386 | 0.241 | 0.377 | 0.212 | | |  |
| **Month 4** | 0.174 | 0.720 | 0.786 | 0.290 | 0.910 | 0.084 | 0.315 | 0.799 | 0.257 | 0.412 | 0.932 | 0.213 | | |  |
| **Month 5** | 0.008 | 0.239 | 0.940 | 0.247 | 0.610 | 0.500 | 0.565 | 0.479 | 0.948 | 0.805 | 0.076 | 0.340 | | |  |
| **Month 6** | 0.391 | 0.233 | 0.569 | 0.491 | 0.511 | 0.051 | 0.726 | 0.677 | 0.381 | 0.484 | 0.069 | 0.222 | | |  |
|  |  |  |  |  |  |  |  |  |  |  |  | |  |  |  |

**Supplementary Table 4.** Oocyst Enumeration per Oocyst Positive Samples. The total number of oocysts calculated per grouping of mosquito dissections in oocyst positive samples. HIV-1 Neg. are the HIV-1 negative samples, and the HIV-1 Pos. ND are the HIV-1 positive newly diagnosed samples.

| **Subset sample** | **HIV-1 group** | **Gametocyte Positive vs Negative (ddPCR)** | **Parasite Positive vs Negative**  **(*18S* qPCR)** | **Number of Mosquitoes Dissected** | **Oocysts per Midgut for Each Dissected Mosquito** | **Total Oocyst**  **Count** |
| --- | --- | --- | --- | --- | --- | --- |
| 1 | HIV-1 Neg. | Negative | Negative | 25 | [0,0,0,0,0,0,0,0,0,0,0,0,0,0,0,0,2,0,0,0,0,0,0,0,0,0] | 2 |
| 2 | HIV-1 Pos. ND | Positive | Negative | 25 | [0,0,3,0,0,0,0,0,0,0,0,0,0,0,0,0,0,0,0,0,0,0,0,0,0] | 3 |
| 3 | HIV-1 Pos. ND | Negative | Positive | 23 | [0,0,0,0,0,0,0,0,0,0,0,0,0,0,0,0,0,0,0,0,0,0,1] | 1 |
| 4 | HIV-1 Pos. ND | Positive | Negative | 25 | [0,6,0,0,0,1,0,0,0,0,0,0,0,0,0,0,0,0,0,0,0,0,0,0,0] | 7 |
| 5 | HIV-1 Neg. | Positive | Negative | 25 | [1,0,0,0,0,0,0,0,0,0,0,3,0,0,0,0,0,0,0,0,0,0,0,0,0,0] | 4 |
| 6 | HIV-1 Neg. | Positive | Negative | 26 | [0,1,0,0,0,0,0,0,0,0,0,0,0,0,0,0,0,0,0,0,0,0,0,0,0,0] | 1 |
| 7 | HIV-1 Pos. ND | Positive | Negative | 26 | [0,0,0,1,0,0,0,0,0,0,0,0,0,0,0,0,0,0,0,0,0,0,0,0,0,0] | 1 |
| 8 | HIV-1 Pos. ND | Positive | Positive | 25 | [0,0,0,0,0,1,0,0,0,0,0,0,0,0,0,0,0,0,0,0,0,0,0,0,0] | 1 |
| 9 | HIV-1 Pos. ND | Positive | Negative | 25 | [0,0,1,0,0,0,0,0,0,0,0,0,0,0,0,0,0,0,0,0,0,0,0,0,0] | 1 |
| 10 | HIV-1 Pos. ND | Positive | Negative | 26 | [0,0,0,0,1,0,0,0,0,0,0,0,0,0,0,0,0,0,0,0,0,0,0,0,0,0] | 1 |
| 11 | HIV-1 Pos. ND | Positive | Positive | 26 | [0,0,0,0,0,0,0,0,0,0,0,0,0,0,0,0,0,0,1,0,0,0,0,0] | 1 |
| 12 | HIV-1 Pos. ND | Negative | Positive | 39 | [0,0,0,0,1,0,0,0,0,0,0,0,0,0,0,0,0,0,0,0,0,0,0,0,0,0,0,0,0,0,0,0,0,0,0,0,0,0,0] | 1 |
| 13 | HIV-1 Pos. ND | Negative | Negative | 39 | [0,0,0,0,0,0,0,0,0,0,0,0,0,0,0,0,0,0,0,0,0,0,0,0,0,0,0,0,0,0,0,0,0,0,0,0,0,1,0] | 1 |
| 14 | HIV-1 Pos. ND | Negative | Negative | 12 | [0,0,0,0,0,0,1,0,0,0,0,0] | 1 |
| 15 | HIV-1 Neg. | Negative | Positive | 25 | [1,0,0,0,0,0,0,0,0,0,0,0,0,0,0,0,0,0,0,0,0,0,0,0,0] | 1 |
| 16 | HIV-1 Pos. ND | Negative | Negative | 26 | [0,0,0,0,0,1,0,0,0,0,0,0,0,0,0,0,0,0,0,0,0,0,0,0,0,0] | 1 |
| 17 | HIV-1 Pos. ND | Positive | Positive | 22 | [0,0,0,8,0,0,0,0,0,0,0,3,0,0,0,0,0,0,0,0] | 11 |
| 18 | HIV-1 Neg. | Negative | Negative | 25 | [0,0,0,0,0,0,0,0,0,0,0,0,0,0,0,0,0,1,0,0,0,0,0,0,0] | 1 |
| 19 | HIV-1 Neg. | Positive | Positive | 25 | [0,4,0,0,0,0,26,0,0,0,0,0,0,0,0,0,0,0,0,0,1,0,0,0,0] | 31 |
| 20 | HIV-1 Pos. ND | Positive | Negative | 24 | [0,0,0,0,0,0,0,0,0,0,0,0,0,0,0,0,0,0,0,1,0,0,0,0] | 1 |
| 21 | HIV-1 Neg. | Positive | Negative | 25 | [0,0,1,0,0,0,0,0,0,0,0,0,0,0,0,0,0,0,0,0,0,0,0,0,0] | 1 |
| 22 | HIV-1 Neg. | Negative | Positive | 27 | [0,0,0,0,0,0,0,0,0,0,0,0,0,0,1,0,0,0,0,0,0,0,0,0,0,0,0] | 1 |
| 23 | HIV-1 Neg. | Negative | Positive | 26 | [0,3,0,0,0,0,0,0,0,0,0,0,0,0,0,0,0,0,0,0,0,0,0,0,0] | 3 |
| 24 | HIV-1 Pos. ND | Positive | Positive | 25 | [0,0,0,0,0,0,2,0,0,0,0,0,0,0,0,0,0,0,0,0,0,0,0,0,0] | 2 |
| 25 | HIV-1 Neg. | Positive | Positive | 26 | [0,0,0,0,0,0,0,0,0,1,0,0,0,0,0,0,0,0,0,0,0,0,0,0,0,0] | 1 |
| 26 | HIV-1 Neg. | Negative | Negative | 24 | [0,0,0,0,0,0,0,0,0,0,0,0,0,0,0,0,0,0,0,0,0,1,0,0] | 1 |
| 27 | HIV-1 Neg. | Negative | Positive | 25 | [0,0,0,0,0,0,0,0,0,0,0,0,0,0,0,0,0,0,0,0,1,0,0,0,0] | 1 |
| 28 | HIV-1 Pos. ND | Positive | Negative | 24 | [0,1,0,0,0,0,0,0,0,0,0,0,0,0,0,0,0,0,0,0,0,0,0,0] | 1 |
| 29 | HIV-1 Neg. | Positive | Positive | 25 | [0,1,0,0,0,0,0,0,0,0,0,0,0,0,0,0,0,0,0,0,0,0,0,0,0] | 1 |
| 30 | HIV-1 Neg. | Positive | Positive | 25 | [0,0,0,0,0,1,0,0,0,0,0,0,0,0,0,0,0,0,0,0,0,0,0,0,0] | 1 |
| 31 | HIV-1 Pos. ND | Positive | Positive | 25 | [0,0,0,0,0,0,0,0,0,0,0,0,0,0,0,0,0,0,0,3,0,0,0,0,0] | 3 |
| 32 | HIV-1 Pos. ND | Positive | Negative | 24 | [0,0,0,0,0,0,0,0,0,0,1,0,0,0,0,0,0,0,0,0,0,0,0,0] | 1 |
| 33 | HIV-1 Neg. | Positive | Positive | 25 | [0,0,1,0,0,0,0,0,0,0,0,0,0,0,0,0,0,0,0,0,0,0,0,0,0] | 1 |
| 34 | HIV-1 Neg. | Positive | Positive | 25 | [0,0,0,0,0,1,0,0,0,0,0,0,0,0,0,0,0,0,0,0,0,0,0,0,0] | 1 |
